# Past climate cooling and orogenesis of the Hengduan Mountains have influenced the evolution of *Impatiens* sect. *Impatiens* (Balsaminaceae) in the Northern Hemisphere

**DOI:** 10.1186/s12870-023-04625-w

**Published:** 2023-11-29

**Authors:** Fei Qin, Tiantian Xue, Xiaoxia Zhang, Xudong Yang, Jianghong Yu, Sudhindra R. Gadagkar, Shengxiang Yu

**Affiliations:** 1 State Key Laboratory of Plant Diversity and Specialty Crops / State Key Laboratory of Systematic and Evolutionary Botany, Institute of Botany, Chinese Academy of Sciences, Beijing, 100093 China; 2 University of Chinese Academy of Sciences, Beijing, 100049 China; 3 China National Botanical Garden, Beijing, 100093 China; 4 Department of Earth System Science, Tsinghua University, Beijing, 100084 China; 5 College of Forestry, Guizhou University, Guiyang, 550025 China; 6 Biomedical Sciences Program, College of Graduate Studies, Midwestern University, Glendale, AZ 85308 USA; 7 College of Veterinary Medicine, Midwestern University, Glendale, AZ 85308 USA; 8 Arizona College of Osteopathic Medicine, Midwestern University, Glendale, AZ 85308 USA

**Keywords:** Balsaminaceae, *Impatiens* sect. *Impatiens*, Ancestral niche, Distribution pattern, Diversification, Phylogenomics

## Abstract

**Background:**

*Impatiens* sect. *Impatiens* is distributed across the Northern Hemisphere and has diversified considerably, particularly within the Hengduan Mountains (HDM) in southwest China. Yet, the infra-sectional phylogenetic relationships are not well resolved, largely due to limited taxon sampling and an insufficient number of molecular markers. The evolutionary history of its diversification is also poorly understood. In this study, plastome data and the most complete sampling to date were used to reconstruct a robust phylogenetic framework for this section. The phylogeny was then used to investigate its biogeographical history and diversification patterns, specifically with the aim of understanding the role played by the HDM and past climatic changes in its diversification.

**Results:**

A stable phylogeny was reconstructed that strongly supported both the monophyly of the section and its division into seven major clades (Clades I-VII). Molecular dating and ancestral area reconstruction suggest that sect. *Impatiens* originated in the HDM and Southeast China around 11.76 Ma, after which different lineages dispersed to Northwest China, temperate Eurasia, and North America, mainly during the Pliocene and Pleistocene. An intercontinental dispersal event from East Asia to western North America may have occurred via the Bering Land Bridge or Aleutian Islands. The diversification rate was high during its early history, especially with the HDM, but gradually decreased over time both within and outside the HDM. Multiple linear regression analysis showed that the distribution pattern of species richness was strongly associated with elevation range, elevation, and mean annual temperature. Finally, ancestral niche analysis indicated that sect. *Impatiens* originated in a relatively cool, middle-elevation area.

**Conclusions:**

We inferred the evolutionary history of sect. *Impatiens* based on a solid phylogenetic framework. The HDM was the primary source or pump of its diversity in the Northern Hemisphere. Orogeny and climate change may have also shaped its diversification rates, as a steady decrease in the diversification rate coincided with the uplift of the HDM and climate cooling. These findings provide insights into the distribution pattern of sect. *Impatiens* and other plants in the Northern Hemisphere.

**Supplementary Information:**

The online version contains supplementary material available at 10.1186/s12870-023-04625-w.

## Background

Mountains cover only about one-eighth of the Earth’s land area, yet they are often associated with a high biodiversity, harboring one-third of global terrestrial species [1–6]. Mountain formation is assumed to be a precondition for rapid speciation, as it creates vicariance, establishes topographic heterogeneity and novel habitats that promote species evolution and diversification [1, 6, 7]. Meanwhile, global cooling at the Mid-Miocene to Pliocene, which intensified in the Pleistocene [8, 9], and the subsequent climatic fluctuations during Pliocene and Pleistocene as well as the climatic change triggered by mountain building in eastern Asia generally resulted in environmental changes. This promoted dispersal of and fragmentation of species distributions and instigated species divergence and new species formation, thus affecting the diversification and distribution of mountain species [10–12]. Moreover, climate change emerged as a major force promoting vegetation dynamics, particularly in the transitions between forests and grasslands, whichs generated successive expansions and retractions of Amazon and Atlantic Forests [13, 14]. Therefore, mountain formation and the consequent climate change have been suggested to account partly for the occurrence of high levels of biodiversity and endemism in mountain areas [12, 15].

The origin of the high biodiversity in mountain regions has been the focus of intense interest [6, 16]. A great deal of work has been done in the Andes [3, 4, 17–19], Alps [20], and the Qinghai-Tibet Plateau (QTP) [10, 21–23] to investigate the potential effects of historical orogenesis and climatic changes on the diversification and distribution of many groups and determine the relative contributions of immigration and in situ diversification to evolution in their biodiversity hotspots. By contrast, the rate and pattern of species diversification in the Hengduan Mountains (HDM) have been somewhat neglected, despite their extraordinary species richness [5, 12, 24]. There appears to be evidence that the HDM may have acted as a primary source area for lineages migrating out and colonizing other parts of the Northern Hemisphere [25]. On the other hand, far fewer numbers of species seem to have immigrated into and colonized the HDM from other regions of the Northern Hemisphere [26, 27]. These differing patterns make the HDM an excellent system for studying the complex biotic interactions within the Northern Hemisphere.

The HDM range is located in southwestern China, at the southeastern margin of the QTP, with an average elevation between 1400 and 5300 m above sea level and a surface area ~ 500,000 km^2^ [28, 29]. The multiple north-south oriented mountain ridges and the dramatic variation in climate and topography provide optimal conditions for allopatric speciation and explain why the HDM form a global biodiversity hotspot [30]. Indeed, the HDM range has one of the world’s richest temperate flora and fauna and a high level of endemism, with ~ 12,800 species of vascular plants (of which at least 26% of the seed plants are endemic) and ~ 1,500 species of terrestrial vertebrates [29, 31, 32]. The uplift of the HDM is thought to have occurred mainly between the late Miocene and late Pliocene, after the formation of the remainder of the QTP [33–36]. However, according to the latest fossil and tectonic evidence, the uplift of the HDM can be traced back to the early Eocene, such that the mountains had reached their current elevation by the early Oligocene, with continued deformation from the late Oligocene to the early Miocene [37–39]. The orogeny of the HDM and associated climate changes have been implicated in the recent floristic diversification and assembly [11, 12, 36].

The species-rich section *Impatiens* includes about 90 species, the majority of which are distributed in subtropical and temperate zones of the Northern Hemisphere, mainly in the mountains of southwestern China; only three species have been identified in Europe and North America [40]. This relatively young group that shows especially high diversity in corolla color and morphology [41, 42] comprises annual or perennial herbs found at elevations ranging from 300 m to as high as 4,200 m above sea level, with habitats including the forest understory, roadside ditches, and along streams [42]. It includes many endemic species with a narrow and fragmented distribution, mostly confined to the HDM, and new species are constantly being discovered [43–47]. Indeed, the HDM and its adjacent areas in Southwest China appear to be the hub for diversity in this section. Approximately one-third of the species in this section occur in the HDM, and almost all are endemic [48]. The unusual distribution pattern and high species richness in these mountains make the HDM an excellent model for exploring the processes responsible for the extraordinary biodiversity of the region. However, while there have been a number of studies on plant diversity in the HDM [12, 31], there has been no comprehensive study with a focus on the biogeography of sect. *Impatiens* in this region.

In their study of the rapid radiation of the *Impatiens* genus during the Pliocene and Pleistocene, Janssens et al. [49] argued that it originated in southwest China and started to diversify in the Early Miocene (albeit with a relative low net diversification rate), which coincided with global cooling of the Earth’s climate and subsequent glacial oscillations. Although these findings shed new light on the origin and evolutionary radiation of *Impatiens*, there is limited information concerning the evolutionary history of sect. *Impatiens*, particularly the role of the HDM in shaping taxon dispersal and in situ diversification, due to inadequate sampling at the sectional level (7 species, corresponding to 7.8% of the species making up the section) and the limited length of the molecular markers used for phylogenetic inference [49]. Furthermore, while previous studies have focused on resolving the phylogenetic relationships at the genus level [40, 50–53], a robust, well resolved phylogenetic framework based on extensive geographic and taxonomic sampling from sect. *Impatiens* has been lacking. Likewise, the ecological processes that shaped the patterns of its spatiotemporal diversity in response to the orogenesis of the HDM and climatic changes, remain largely unexplored.

In this study, plastomes and the most comprehensive sample of sect. *Impatiens* to date were used to reconstruct a highly robust phylogeny tree, resulting in tree topologies with the highest confidence values to date. This phylogenetic framework was applied to investigate the divergence times, biogeographic history, diversification rate, and ancestral niches of the section, with the aim of determining the role played by the HDM and past climatic changes in shaping its spatiotemporal evolution. Specifically, we examined the role of mountain formation and paleoclimate on the drivers of diversification and taxon assembly within the HDM and in the evolutionary history and diversification of sect. *Impatiens*.

## Results

### Phylogenomic analysis of sect. *Impatiens*

**Fig. 1:**
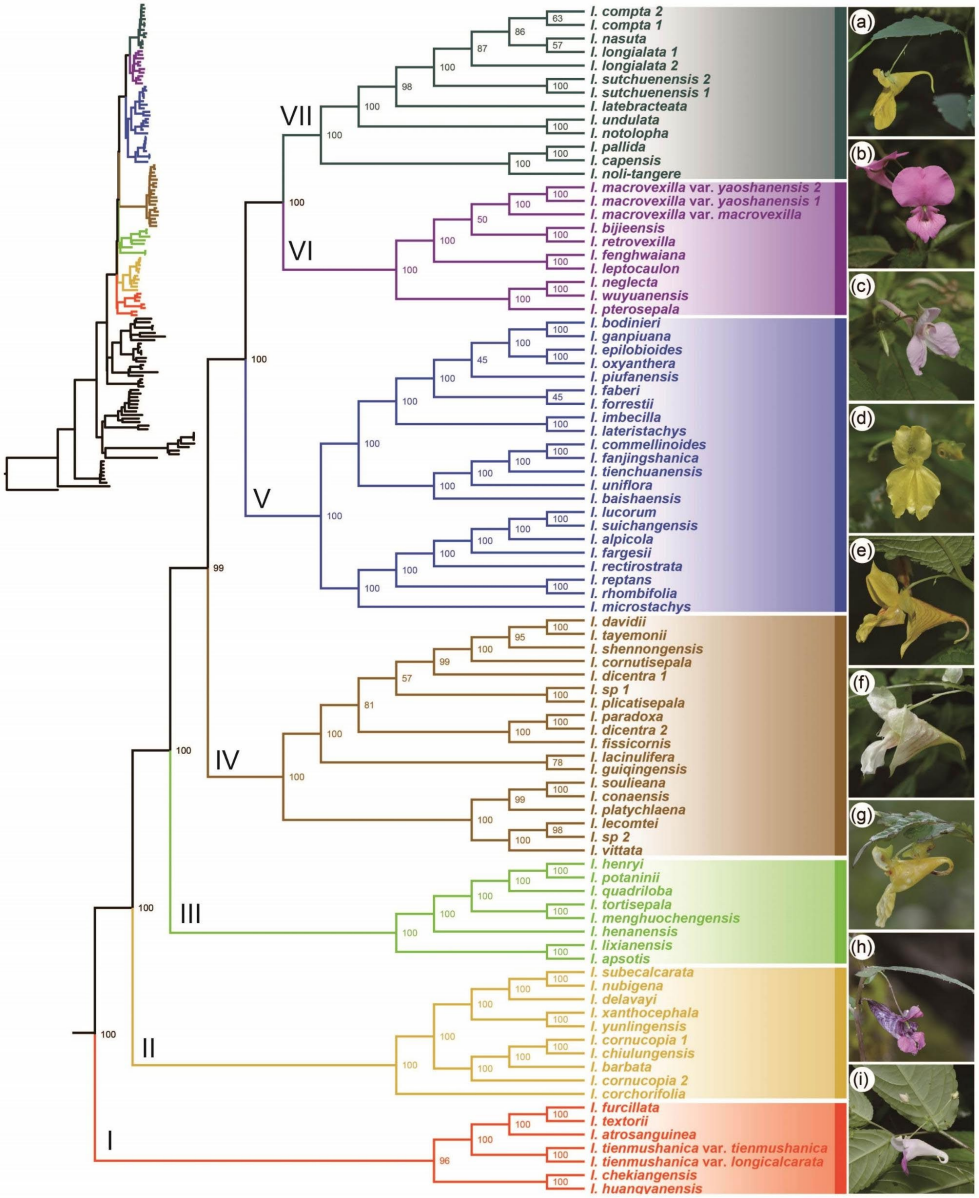
Maximum likelihood tree of *Impatiens* sect. *Impatiens* inferred from concatenated nucleotide sequences of 80 plastid coding genes. Numbers above the branches are bootstrap values. Photographs of representative species on the right: (**a**) *I*. *noli–tangere*, (**b**) *I*. *macrovexilla* var. *yaoshanensis*, (**c**) *I*. *imbecilla*, (**d**) *I*. *alpicola*, (**e**) *I*. *soulieana*, (**f**) *I. vittata*, (**g**) *I*. *tortisepala*, (**h**) *I*. *delavayi*, and (**i**) *I*. *tienmushanica*. Photographs are by D–H Zhu and S–X Yu

Based on the matrices of whole plastomes and a concatenation of 80 plastid coding genes (CDSs), both maximum likelihood (ML) and Bayesian inference (BI) methods generated well resolved, highly congruent tree topologies (Fig. 1, Figs. S1-S3). Strong support was obtained for the monophyly of sect. *Impatiens*, and its phylogenetic relationships with the outgroups were well resolved. Seven main clades (Clades I-VII) were recovered robustly, with high bootstrap support in the ML tree (BS = 96%/100%) and posterior probability in the Bayesian tree (PP = 1). Clade I is sister to the remaining taxa of the section, followed by Clades II to VII; Clade III was identified for the first time. The internal relationships within Clades I-III were all resolved with strong support (BS = 100%, PP = 1). However, despite moderate or strong support for most of the nodes, the phylogenetic positions of a few species were not well resolved in Clades IV-VII.

Within Clade I, Northeast Asian species clustered together with strong support; *I*. *atrosanguinea* and the clade (*I*. *furcillata*, *I*. *textorii*) were strongly supported as sisters. Clade III, recovered for the first time, consisted of eight species and all nodes received the strongest support. Within Clade IV, the internal relationships received moderate or strong support, and two samples of the same species (*I*. *dicentra*) were polyphyletic. Three subclades were seen in Clade V, and all of the phylogenetic relationships were well resolved with the strongest support, except for two nodes. Within Clades VI and VII, multiple samples of the same species clustered together (*I*. *macrovexilla* var. *yaoshanensis*, *I*. *sutchuenensis*, and *I. compta)*. Within Clade VII, the sister grouping of the North American species (*I*. *pallida*, *I*. *capensis*) was strongly supported (BS = 100%, PP = 1), and these in turn, were sister to *I*. *noli-tangere*, a species widespread in the temperate zone of the Northern Hemisphere (BS = 100%, PP = 1).

### Divergence time estimation

The divergence times estimates from BEAST yielded stem and crown ages for sect. *Impatiens* of ca. 16.15 Ma (95% highest posterior density [HPD]: 10.84–21.22 Ma, Fig. S4) and ca. 11.76 Ma (95% HPD: 8.14–16.08 Ma) during the early Miocene and middle Miocene, respectively. Clade I began to diversify ca. 11.35 Ma (95% HPD: 7.29–14.99 Ma) in the late Miocene. The estimated stem ages of Clade II and Clade III were ca. 11.45 Ma (95% HPD: 7.74–15.40 Ma) and 11.04 Ma (95% HPD: 7.52–14.91 Ma) during the late Miocene, respectively. Clade IV originated ca. 10.73 Ma (95% HPD: 7.06–14.3 Ma) in the late Miocene and began to diversify ca. 4.39 Ma (95% HPD: 2.93–6.04 Ma) in the early Pliocene. The stem age of Clade V dated back to the late Miocene (ca. 8.56 Ma, 95% HPD: 5.64–11.51 Ma). The split between Clade VI and Clade VII occurred ca. 6.23 Ma (95% HPD: 4.14–8.58 Ma), during the late Miocene, and the onset of diversification in Clade VI and Clade VII ca. 4.68 Ma (95% HPD: 3.00-6.26 Ma) and 2.71 Ma (95% HPD: 1.86–3.75 Ma) in the Pliocene, respectively.

### Ancestral area reconstruction

**Fig. 2:**
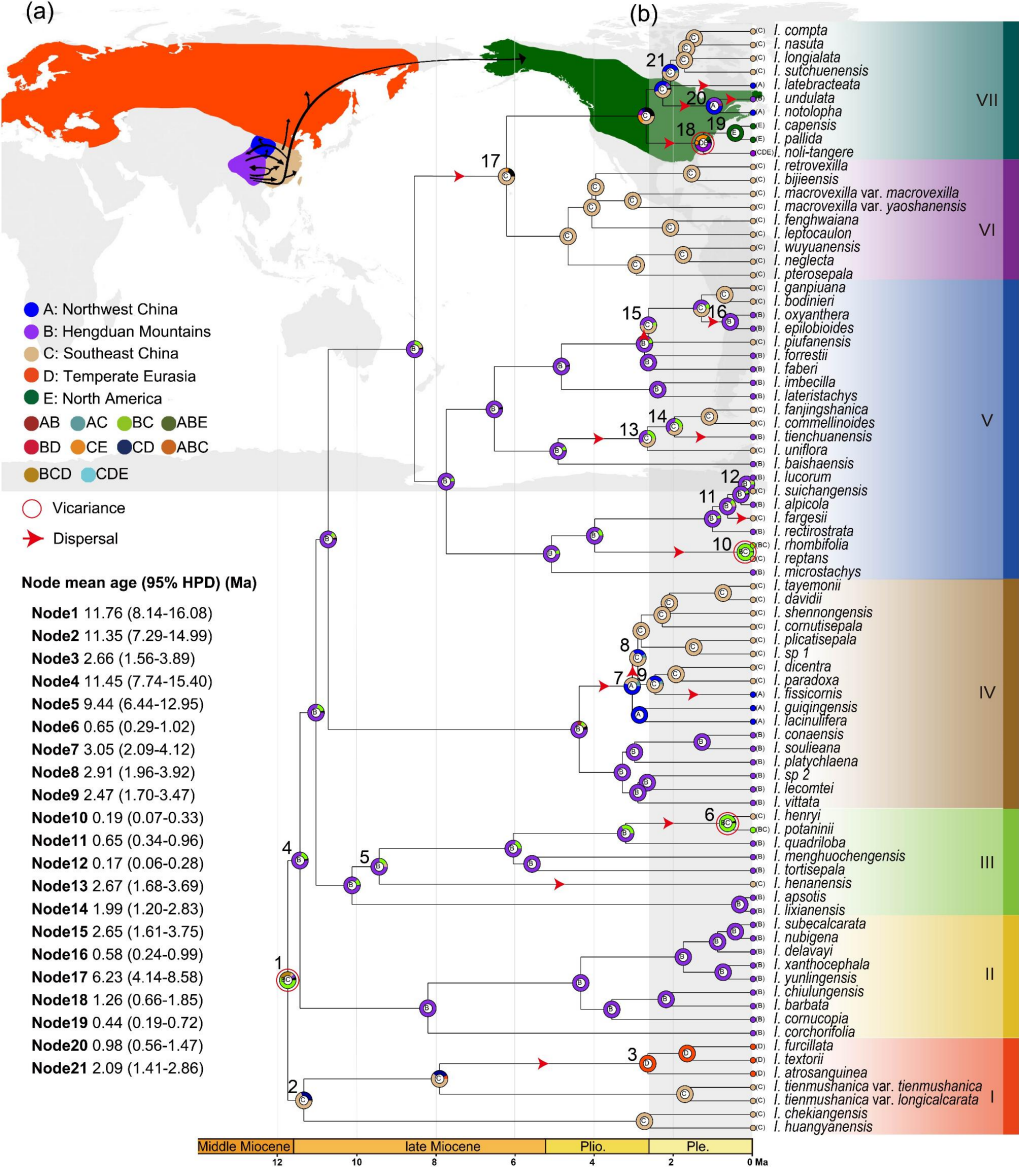
Estimation of the biogeographic history of *Impatiens* sect. *Impatiens*. (**a**) Potential dispersal routes of *Impatiens* sect. *Impatiens*. (**b**) Ancestral range estimation was done using BioGeoBEARS with the DEC + J model and is shown for each ancestral node by means of colored rings and the corresponding letter(s). Vicariance events are indicated by red circles around the internal nodes and dispersal is shown by red arrows along the branches. Ancestral age estimation (mean and 95% HPD) was done and is shown for the numbered nodes on the left. The map used in this study was downloaded from DIVA-GIS (http://www.diva-gis.org/Data)

BioGeoBEARS returned the model DEC + J, which had the lowest AICc, as the best model (Table S2). Ancestral area reconstruction showed that the most recent common ancestor (MRCA) of sect. *Impatiens* most likely was present in the HDM and Southeast China (Fig. 2, node 1, BC = 0.55), with multiple dispersal events to Northwest China, temperate Eurasia, and North America. In the early history of sect *Impatiens*, a vicariance event (BC->B/C) may have occurred, resulting in two branches. It appears that the MRCA of Clade I was present in Southeast China (node 2, C = 0.69), while the MRCA of the rest of the species existed and subsequently diversified within the HDM itself (node 4, B = 0.79). Within Clades II-VII, the MRCA of four main clades (Clades II-V) was inferred to have been in the HDM; however, in the node 17 the MRCA of Clades VI and VII was retrieved with ancestral distribution in Southeast China (node 17, C = 0.73). This distribution is possible with a dispersal event from HDM to Southeast China (node 17).

The MRCA of Clade I existed and diversified in Southeast China and then colonized temperate Eurasia (Northeast Asia), at 2.66 Ma (95% HPD: 1.56–3.89; node 3). Within Clade IV, successive dispersal events from the HDM to Northwest China and Southeast China likely occurred, dating to 3.05 Ma (95% HPD: 2.09–4.12; node 7) and 2.91 Ma (95% HPD: 1.96–3.92; node 8). The MRCA of Clade II was in the HDM and underwent in situ diversification, and no dispersal events could be inferred, just as for Clades VI in Southeast China. On the other hand, the MRCA of Clades III and V was distributed in the HDM and subsequently dispersed into Southeast China several times during the late Pliocene-Pleistocene (ca. 2.67 − 0.19 Ma), with those within Clade V even re-emigrating back into the HDM at 0.58 Ma (95% HPD: 0.24–0.99; node 16). A dispersal event from Southeast China to the temperate Eurasia and North America at 1. 26 Ma (95% HPD: 0.66–1.85) was inferred for Clade VII (node 18). Thereafter, the clade appears to have diverged, through geographical isolation, to form the North American endemic clade (*I*. *pallida*, *I*. *capensis*) (node 19). Besides, within Clade VII, two dispersal events from Southeast China to Northwest China (node 20), and one remigration event from Northwest China to the HDM were inferred.

### Diversification rate analyses

**Fig. 3:**
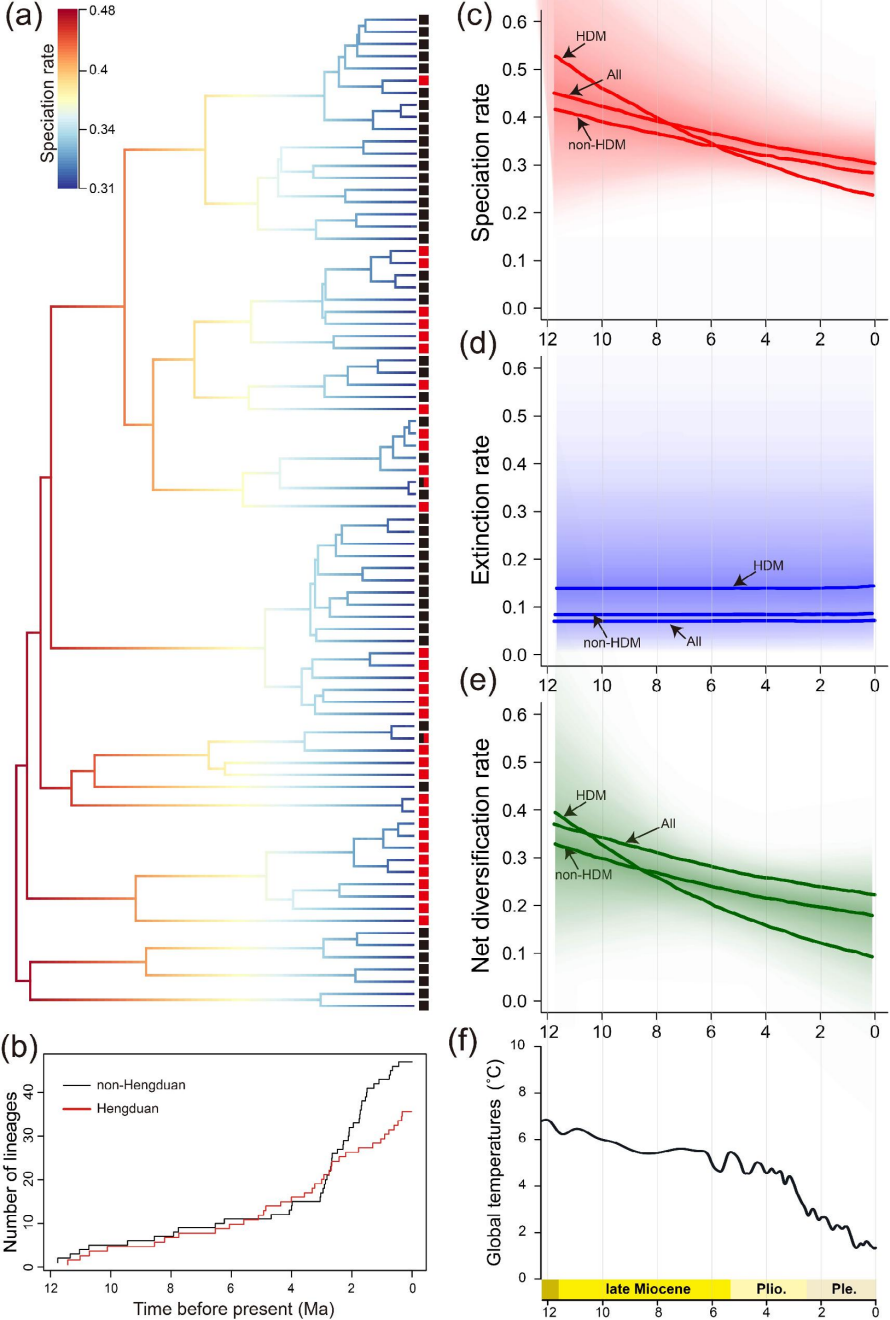
Analysis of diversification rate of *Impatiens* sect. *Impatiens*. (**a**) Variation in speciation rates across the phylogeny of the section. Species distributed in the Hengduan Mountains (HDM) are indicated with red squares, and those in other areas are indicated with black squares. (**b**) A lineage-through-time plot for sect. *Impatiens* within the HDM and non-HDM. (**c**-**e**) Temporal variation in speciation, extinction, and net diversification rates inferred with a Bayesian analysis of macroevolutionary mixtures. (**f**) Temperature fluctuations since the late Miocene [12]

According to the lineage-through-time (LTT) plot, sect. *Impatiens* underwent constant species diversification during the late Miocene, with a surge in lineage accumulation after ca. 3 Ma (Fig. S5). Bayesian analysis of macroevolutionary mixtures (BAMM) showed that the speciation rate (λ) and net diversification rate (*r*) were highest at 11.76 Ma (λ = 0.46 species/Ma, *r* = 0.38 species/Ma) but then decreased over time, whereas the extinction rates remained constant (μ = 0.08 species/Ma) (Fig. 3). The regional LTT results showed that lineage accumulation in both regions was initially slow and relatively constant, with acceleration occurring at approximately ca. 3.4 Ma (HDM) and 2.9 Ma (non-HDM), during which, accumulation in the non-HDM exceeded that in the HDM after ca. 2.5 Ma (Fig. 3B). The BAMM also suggested an initially higher speciation rate (λ) and net diversification rate (*r*) within the HDM than that outside the HDM, but also a greater decrease in the speciation/net diversification rate within than outside the HDM. Furthermore, the extinction rates within and outside the HDM remained constant, while the extinction rate was higher within than outside the HDM (Fig. 3). Among the seven clades, the speciation rate increased over time only in Clade II; in Clade I it remained almost constant, while in the remaining five clades it decreased (Fig. S6).

### Distribution of species richness, and the ecological drivers of diversification

Distribution pattern analysis showed that sect. *Impatiens* was largely confined to East Asia, North America, and Europe, consistent with a Northern Hemisphere distribution pattern. However, diversity hotspots were confined to the HDM and the Bashan-Wushan Mountains (Fig. S7). The first and second axes of the principal components analysis (PCA) together explained 57.5% of the total variance (Figs. S8, S9). The first axis explained 38.4% of the total variance, correlated with the mean temperature of the driest quarter (BIO9), the annual mean temperature (BIO1), and temperature seasonality (BIO4). The second axis explained 19.1% of the total variance, mainly describing the mean diurnal temperature range (BIO2) and the mean temperature of the wettest quarter (BIO8).

**Fig. 4:**
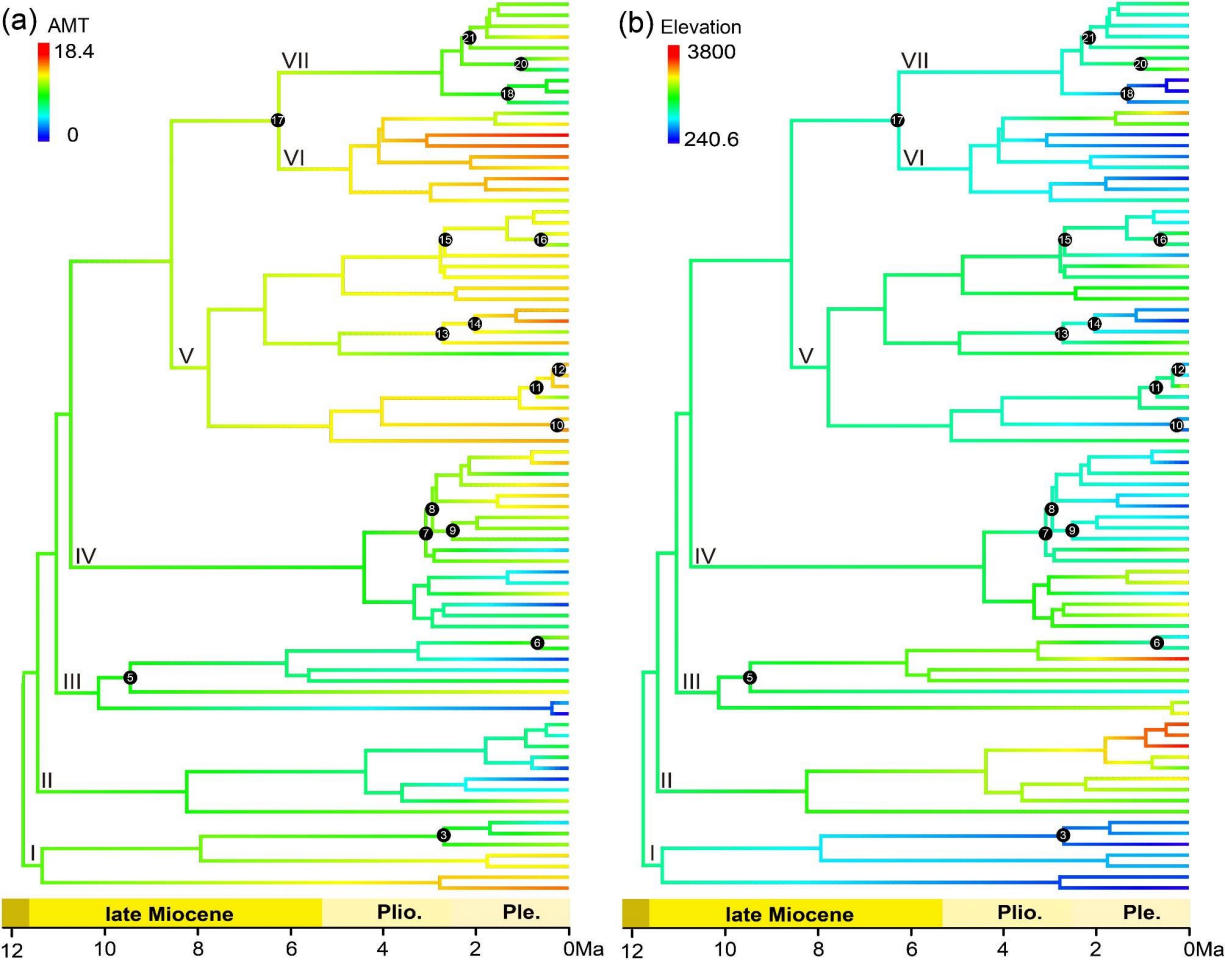
Ancestral niche reconstructions of *Impatiens* sect. *Impatiens*. (**a**) Annual mean temperature. (**b**) Elevation. Branch numbers indicate the node number (see Fig. 2)

In the multiple linear regression of each environmental factor vs. species richness, the adjusted R-squared of the model was 0.306 (Table S3). The species richness of the section was significantly associated with six environment variables, four of which [elevation range, BIO1, annual precipitation (BIO12), and elevation] were positively correlated with species richness; the other two [isothermality (BIO3) and precipitation in the driest month (BIO14)] were negatively correlated with species richness. Among these variables, elevation range (22.40%) was the most important, followed by BIO1 (18.32%) and elevation (16.70%). The annual mean temperature and elevation niches for the MRCA of sect. *Impatiens*, as reconstructed, were 10.91℃, and 1964 m, respectively (Fig. 4), suggesting that the section originated in a relatively cool, middle-elevation area. Among the 18 estimated dispersal events, eight consisted of spreading to warmer areas and 15 involved moving to lower elevations areas.

## Discussion

### Phylogeny of sect. *Impatiens*

This study has, for the first time, produced a highly robust phylogenetic framework of the sect. *Impatiens*, with the help of the largest taxon sample (82/90), 80 CDSs (Fig. 1, S1 and S2), and whole plastomes (Fig. S3). The topologies built from the two datasets were mutually congruent and the inferences were consistent with the conclusions of other studies [40, 49–52, 54]. The systematic position of the section has previously been debated, apparently due to the limited number of molecular markers [40, 54]. However, we were able to confirm the sister relationship between sect. *Impatiens* and the clade formed by sect. *Fasciculatae* plus sect. *Scorpioidae* and sect. *Uniflorae*, consistent with a previous phylogenomic study of *Impatiens* [53]. The internal phylogenetic relationships of the section were also well resolved, with strong support for seven major clades (Clades I-VII) recognized based on the plastome data (Fig. 1, Figs. S1-S3). These results provide a solid framework for further studies on sectional spatiotemporal and macroevolutionary patterns.

The greater breadth of our taxon sampling revealed a new clade, Clade III, with strong support and unambiguous internal relationships. In previous studies, perhaps due to the low resolution of molecular markers and limited sampling based on only two taxa from this clade, namely, *I. apsotis* and *I. tortisepala*, the phylogenetic positions of these two species were incongruent such that they were not considered to be part of a separate clade [40, 50, 51, 54]. Extensive sampling in our study has also allowed the major clades to be re-circumscribed, as the ambiguous phylogenetic positions of several species were resolved. For example, *I*. *atrosanguinea*, once considered a synonym of *I*. *textorii* [55], was shown to have a sister relationship with the clade made up of the latter and *I*. *furcillata* (Clade I; Fig. 1). In our study, *I*. *davidii* was resolved as a member of Clade IV, which contradicts the studies by Yuan et al. [50] and Ruchisansakun et al. [56], but agrees with those of Yu et al. [40] and Song et al. [54]. This discrepancy was also likely due to the sequence data in the previous studies being made up of fewer molecular fragments. Our study is empirical proof that the prerequisites to reconstructing a robust phylogenetic framework are highly representative sampling with accurate nomenclature and adequate (molecular) data.

### Divergence times and biogeography of sect. *Impatiens*

Previous studies indicated that an increase in sampling representativeness can provide a more accurate estimation of the divergence time [57, 58], such that the time obtained in this study is likely to be more reliable. Our estimate puts the time of the initial diversification of this section much earlier (11.76 Ma; 95% HPD: 7.99–13.03) vs. 4.96 Ma (SD = ± 1.1) than a previous study [49] although we used the same secondary calibration points. The difference can again be attributed to the much higher representativeness of our sampling, both at the level of the taxa and the molecular data. Moreover, our biogeographic analyses indicate that the section originated in the HDM and Southeast China, contradicting Janssens et al. [49], who argue that the MRCA of sect. *Impatiens* occurred in Southwest China. Janssens et al. [49] also reported a dispersal event from Southwest China to North America via the Bering land bridge during the Pleistocene (1.32–1.27 Ma). We inferred a dispersal event from Southeast China to temperate Eurasia and North America ca. 1.26 Ma (95% HPD: 0.66–1.85), followed by the formation of a North American clade through a vicariance event. Given that *I*. *noli-tangere* is the only transcontinental species, covering Northeast Asia, Europe, and western North America, we argue that the geographic expansion of sect. *Impatiens* may have occurred via the Bering land bridge (BLB) or Aleutian Islands. The BLB emerged many times during Quaternary ice ages, which would have allowed the successful spread of the species [59]. Moreover, the partial salt resistance and buoyancy of *Impatiens* seeds [60, 61] may have ensured their viability as they were transported by the current across the Bering Strait or Aleutian Islands.

Importantly, our study also revealed that the HDM played an important role in driving the spatiotemporal distribution of sect. *Impatiens* in East Asia. This was achieved by dividing the distribution of the section into four geographical regions, followed by an in-depth analysis of the history of the section’s migration and diffusion in East Asia and the impact of geological events on the distribution pattern. We were thus able to determine that the section originated in the HDM and Southeast China, with the allopatric speciation of the two sister lineages resulting in each inheriting distinct portions of the ancestral range: the MRCA of Clade I in Southeast China and the MRCA of Clades II-VII in the HDM. The latter clades diversified and then migrated to other areas in the Northern Hemisphere multiple times independently. The HDM and non-HDM lineages are intercalated in the phylogenetic tree, indicating that dispersal out of the HDM took place in parallel from different geographic regions at various times. This pattern is similar to that reported for other groups, such as *Polygonatum* Mill. (Asparagaceae) [62] and *Caragana* Fabr. (Leguminosae) [63], both of which originated in the HDM and diverged elsewhere in the Northern Hemisphere. Our results also suggest three colonization events from the non-HMD to the HDM during the Pleistocene (1.99 − 0.58 Ma) and that dispersal between the HDM and neighboring areas was bidirectional, slightly different from the often-reported biogeographical scenario of ‘out of the HDM’ [10, 64–67]. However, the low diversification of the lineages that colonized the HDM suggests that the main mode of speciation of sect. *Impatiens* in the HDM was in situ diversification. The presence of multiple dispersal channels enabled exchanges between the HDM and neighboring areas, such that the colonization of Southeast China by populations dispersed from the southern HDM could be traced back to the late Miocene (6.23 Ma, 95% HPD: 4.14–8.58), and the colonization of Northwest China from the northern HDM occurred in the late Pliocene (3.05 Ma, 95% HPD: 2.09–4.12). Lineages that dispersed from the ancestral region also exchanged with each other (Fig. 2), such as dispersal from Southeast China to Northwest China (node 20) and vice versa (node 8).

### Orogeny and climate change shaped the evolution of sect. *Impatiens* in the Northern Hemisphere

Orogeny led to a vicariance event in the early history of sect. *Impatiens* and promoted the high early diversification rate, while decreasing the diversification rate over time, especially in the HDM. Our results indicate that the basal divergence of the section was around 11.76 Ma (95% HPD: 8.14–16.08 Ma), coinciding with the orogenesis of the southeastern QTP during the Late Miocene [6, 25, 68]. Some other taxa occurring in the HDM and adjacent regions have been similarly estimated to have originated or diverged from their related groups at this time, such as *Rheum* L. (12.0 Ma, 8.2–16.1; Polygonaceae) [69], *Meehania* Britt. ex Small et Vaill. (11.88 Ma, 8.40–16.10; Labiatae) [70], and *Dontostemon* Andrz. ex Ledeb. (11.71 Ma, 7.12–16.83; Cruciferae) [71]. The results of ancestral ecological niche reconstructions analyses also showed that Clade I in Southeast China became adapted to low elevations, whereas Clades II-VII, mostly in the HDM, preferred high-elevation areas (Fig. 4B). This suggests that early allopatric speciation due to the elevation difference (likely related to mountain formation) was a driver of the initial lineage divergence within sect. *Impatiens*. Furthermore, our analysis reveals that the HDM lineages diversified significantly faster than the non-HDM lineages during this period, suggesting that the uplift of mountains and increased topographic complexity may lead to higher diversification rates compared to non-mountain regions. Such a pattern has been shown in Liolaemidae within Andes [3] and *Oreocharis* Benth. (Gesneriaceae) within the HDM [12]. Besides, multiple linear regression analysis also showed that elevation range has a positive relationship to species richness of this section and had the stronger effect than other variables. Therefore, we postulate that orogeny of HDM created a variety of environmental conditions resulting in altitude gradient difference that promoted speciation of sect. *Impatiens*. The rapid early diversification of the section, with a net diversification rate of 0.38 species/Ma, is higher than that of many radiation plant groups in the HDM, such as tribe Anastaticeae (0.17 species/Ma; Brassicaceae) [72] and 17 other angiosperm clades (ca. 0.12 species/Ma) [36], while it is slower than rates detected in other mountains areas [16], for example, *Hypericum* L. within the Andean Mountains (2.5–3.72 species/Ma; Hypericaceae) [73], *Androsace* L. within the Alps Mountains (0.62 species/Ma; Primulaceae) [74].

However, the diversification rate appears to have gradually decreased, particularly in the HDM (Fig. 3c). Thus, while early rapid speciation was likely trigged by orogeny, which drove the exploitation of new ecological niches, diversification occurred at a slower rate and without a corresponding increase in the extinction rate once the available niche space became saturated [12, 75, 76]. However, given that the ancestors of the section likely originated in a relatively cool, middle-elevation area, the steady decline of diversification can be attributed to the further uplift of the HDM during the late Miocene and Pliocene together with the climate cooling that began in the Mid-Miocene. For Clade II, with in situ diversification within the HDM, the diversification rate has continued to increase over time, from 8.2 Ma to the present (Fig. S6). We argue that although the habitat heterogeneity resulting from mountain formation and climate cooling promoted speciation to a certain extent, it decreased the diversification rate of the section as a whole.

Climate cooling since the late Miocene may be the main reason for the decreased diversification rate of sect. *Impatiens*. The PCA analysis showed that the first and second axis mainly describe the temperature-related variables, and multiple linear regression analysis also showed that BIO1 was the second most important variable after elevation range, indicating that temperature is an important variable to shape the distribution pattern of sect. *Impatiens*. Previous studies have highlighted that global temperatures cooling during the middle Miocene, causing subtropicl and warm-temperate elements to retreated southernward, which opened up niches for herbaceous lineages [77, 78]. Furthermore, climatic oscillations during the Pleistocene and Pliocene promoted the diversification of tropical and subtropical species, particularly in mountainous areas, as it has the potential to act as refugia for biodiversity through time [49, 79–81]. As one of the important elements in subalpine, lineage accumulation of sect. *Impatiens* increased exponentially after ca. 3 Ma, particularly within the non-HDM ca. 2.9 Ma. Therefore, climate cooling affected speciation, especially in the non-HDM.

The evolutionary history of the HDM and non-HDM are different, although the diversification rates in both decreased over time. The HDM and Bashan-Wushan areas are the diversity hotspots of sect. *Impatiens.* However, the diversification rate of the lineages within the non-HDM initially decreased slowly but later at a higher diversification rate than of those within the HDM. During the Quaternary climatic oscillations, the topographic complexity of the Bashan-Wushan and Nanling mountains may have provided a buffer against extinction [82] such that the relatively high diversification rate in the non-HDM was maintained. Many lineages originated during this period through allopatric speciation, triggered by those oscillations and the resulting increase in topographic heterogeneity. In contrast, the continuous orogeny and further uplift of the HDM formed the high altitude and low temperature areas, making it more vulnerable to global cooling and unfavorable for the survival of sect. *Impatiens* which prefers warm temperatures. This explains the slower decrease in the diversification rate in non-HDM when compared to that within the HDM. Therefore, the HDM and non-HDM have similar species richness of sect. *Impatiens* today, even though the diversification rate within HDM was higher than non-HDM in its early evolutionary history. Similar patterns have been observed in the Sigmodontinae within Andes [83], *Gammarus lacustris* Sars within Tianshan [8], and *Oreocharis* within the HDM [12].

A dispersal event from East Asia to North America during the Pleistocene led to the emergence of the widespread species, distributed in Europe, East Asia, and North America, whereas most of the species in the section have rather narrow ranges. Polyploidization promotes adaptation to extreme environments [84]. However, the species of sect. *Impatiens* distributed in North American are diploid [85]. The ability of species distributed in North America, including *I*. *noli-tangere*, to acquire a wide distribution range in the Northern Hemisphere despite cooling global temperatures and climatic oscillations, may be due to their reproductive strategy of cleistogamy [86–88]. Our study obtained evidence of multiple dispersal events from the HDM into neighboring lower elevations and relatively warm areas, during the Pliocene and Quaternary. These events may have been driven by the cooling climate and climatic oscillations characteristic of this period [8, 10]. Members of the North American clade of sect. *Impatiens* may be able to adapt to low temperatures, unlike the majority of the species of the section, which prefer warm temperatures. Accordingly, during the uplift of the HDM, species would have dispersed from high elevations to the relatively warm, lower elevation of neighboring areas, where they would have subsequently diversified. In this way, the HDM would have acted as a source or pump of sect. *Impatiens* diversity in the Northern Hemisphere during the Pliocene and Pleistocene, similar to other mountains such as the Andes [89], serving as important biogeographical source and sink for plant interchange.

## Conclusions

Here, plastome data were used to obtain a well-resolved phylogeny for sect. *Impatiens* with extensive taxon sampling. This section was found to be monophyletic and divided into seven clades, among which Clade III was newly identified. The results of molecular dating and ancestral area reconstruction analysis revealed that the section originated in the HDM and Southeast China during the middle Miocene and then dispersed into Northwest China, temperate Eurasia, and North America. Analyses of the diversification rate and ancestral niche reconstructions strongly indicated that climatic fluctuations and the uplift of the HDM during the Pleistocene account for its distribution across the Northern Hemisphere. These results provide new insights into the phylogeny and biogeography of the sect. *Impatiens*, which also enriches important ecological and evolutionary knowledge regarding diversification affected by mountain formation and paleoclimate change.

## Materials and methods

### Taxon sampling, genome-skimming sequencing, plastome assembly, and annotation

Of the 137 plastomes representing 126 taxa analyzed in this study, 119 were newly sequenced and 18 were downloaded from GenBank (Table S1). The dataset included 82 taxa (approximately 90% of estimated species diversity) of sect. *Impatiens* and 44 taxa from outgroups. The outgroups included closely related sections or subgenera, such as subg. *Clavicarpa* (7 taxa), sect. *Semeiocardium* (6), sect. *Racemosae* (12), sect. *Fasciculatae* (1), sect. *Scorpioidae* (4), sect. *Uniflorae* (12), and the more distantly related outgroups *Hydrocera triflora* (L.) Wight & Arn. and *Marcgravia coriacea* Vahl, chosen based on previous studies [40, 50]. The sect. *Impatiens* has an intercontinental distribution across North America and Eurasia, and our sampling covered its geographic range, including all of the main recognized clades reported in previous studies [40, 50, 51, 54]. The formal identification of these samples was undertaken by Shengxiang Yu (Institute of Botany, Chinese Academy of Sciences). All voucher specimens were deposited in the herbarium of the Institute of Botany, Chinese Academy of Sciences. Taxon names, voucher information, and GenBank accession numbers are listed in Table S1.

Total genomic DNA was extracted from silica-dried leaf materials using a modified CTAB method [90]. All samples were sent to Novogene (Beijing, China) for genomic library construction and Illumina sequencing. Before the plastomes were assembled, the raw sequence reads were subjected to quality control by Novogene using fastq software [91], to obtain high-quality clean reads. *De novo* plastome assembly was implemented in the GetOrganelle pipeline [92] with default parameters. For species with contigs only in the GetOrganelle outfile, Bandage v. 0.8.0 [93] was used to manually assemble the plastomes. The draft of the plastome annotation was generated using the ‘Map to Reference’ function in GENEIOUS Prime v.2020.0.5 [94], with the genome of *I*. *fanjingshanica* Y. L. Chen (NC_059944) as reference. The presence of start and stop codons in each plastid coding genes (CDSs) was checked and adjusted manually.

### Phylogenetic analyses

Two datasets (whole plastomes and a concatenated matrix of 80 CDSs) were generated and used for phylogenetic reconstruction. The whole plastomes were aligned using MAFFT v.7.490 [95], and poorly aligned regions were removed using trimAL v. 1.4 [96]. Eighty plastid coding genes (CDSs) were extracted from plastomes and were aligned using MAFFT v.7.490 [95]. After removing the poorly aligned regions, the 80 CDSs were concatenated end-to-end to form a concatenated matrix using Phylosuit v1.2.2 [97]. Phylogenetic analyses of the concatenated dataset of the 80 CDSs were performed using Bayesian inference (BI) and maximum likelihood (ML) methodologies in MRBAYES v.3.2.7 and RAxMLv.8.2.12 on the CIPRES Science Gateway (https://www.phylo.org/), respectively. The best-fit substitution model was determined by the Akaike information criterion (AIC) in jModelTest v.2.1.4 [98]. In the ML analysis, the GTR + G substitution model was assigned, with 1000 bootstrap replicates. For the BI analysis, the GTR + I + G model was assigned, and one tree was sampled every 1000 generations for 2 × 10^6^ generations. In addition, a phylogenetic tree was constructed based on the whole plastome dataset, using ML in IQ-TREE v1.6.15 [99], with 1,000 replicates of the SH-like approximate likelihood-ratio test (SH-aLRT) and the ultrafast bootstrapping algorithm (UFBoot). The best-fitting model was automatically selected by the parameter –m MPF + MERGE.

### Divergence time estimation

As there are presently no known reliable fossils of the Balsaminaceae family (Paleobiology Database, accessed on March 10, 2023) [49], secondary calibrations based on Janssens et al. [49] were used to calibrate a molecular clock and estimate the divergence times of lineages within Balsaminaceae. In their study [49], 11 external fossil calibration points or secondary calibration points were used to estimate the divergence times of lineages within Balsaminaceae, which showed a crown age of *Impatiens* of 22.5 Ma (SD = ± 5.6) and the crown and stem ages of 30.7 Ma (SD = ± 8.6) and 48.2 Ma (SD = ± 9.3) for the Balsaminaceae, respectively. These three age estimates (the crown and stem ages of the Balsaminaceae and the crown age of *Impatiens*) were used as secondary calibration points in our study.

Divergence times were estimated using BEAST v.1.8.4 [100], based on the matrix of concatenated 80 CDSs. In the following analysis, only one sample was retained for each taxon, as shown in Figure S4. First, the DNA substitution model was set to the GTR + I + G model. The uncorrelated log-normal relaxed clock model was assumed as the molecular clock model under the Yule tree prior. The normal priors were set for three secondary calibration points above. Each chain was run for 100 million generations, with sampling every 10,000 generations. We generated the XML file using BEAUTi version 1.8.2 [100] and the divergence dates were estimated using BEAST version 1.8.4 [100]. Five independent runs (with different random starting seeds) were executed in BEAST and the results were combined in Logcombiner version 1.8.2 [100]. Convergence and effective sample sizes > 200 were assessed using Tracer v.1.6 [101]. Finally, the first 25% of trees were discarded as burn-in and the maximum clade credibility (MCC) chronogram was generated from the remaining trees, with the mean node ages and 95% HPD calculated in TreeAnnotator v. 1.8.2 [102].

### Ancestral area reconstruction

Based on the distribution and endemism of sect. *Impatiens*, five geographical regions were defined to cover the distribution of all taxa of the section (Fig. S10): Northwest China, Hengduan Mountains, Southeast China, temperate Eurasia, and North America. The presence or absence of each species in these geographical regions was coded according to an in-house database comprising species distribution information. Ancestral areas were estimated using BioGeoBEARS [103], implemented in RASP v.4.2 [104], applying the MCC tree produced on BEAST. The following models were tested: dispersal-extinction-cladogenesis (DEC), the likelihood version of dispersal-vicariance (DIVALIKE), and the likelihood version of the BayArea model (BAYAREALIKE), including (or not) a “jump dispersal” parameter (J) that permits founder-event speciation in the history of that species. The Akaike information criterion corrected for small sample size (AICc) was used to evaluate the fit for the different models. A maxareas = 3 criterion was used based on the current distribution of the section.

### Diversification rate analysis

Because our focus was on the sect. *Impatiens*, multiple sampling of the same species and outgroups were removed from the MCC tree obtained in the divergence dating analysis. This resulted in the retention of a phylogenetic tree of 82 samples (taxa) of the section, which were used to test the temporal dynamics of diversification. These were visualized by generating a lineage-through-time (LTT) plot of the section using the function “ltt.plot” in the R package “ape” v. 5.3 [105] to plot 7,000 trees (after 30% burn-in) from the data analysis as well as the MCC tree.

In addition, Bayesian analysis of macro-evolutionary mixtures (BAMM) was used to estimate the dynamics of the speciation and extinction rates over time and among lineages. The “globalSamplingFraction” in the control file was specified as 0.9, corresponding to 90% sampling of sect. *Impatiens*, and the Markov chain Monte Carlo (MCMC) was run for 10 million generations and sampled every 5,000 generations. Prior distributions of speciation (λ) and extinction (μ) rates were estimated with the “setBAMMPriors” function in the R package ‘BAMMtools’ version 2.1.6 (ExpectedNumberofShifts = 1.0, lambdaInitPrior = 0.562, lambdaShiftPrior = 0.110, muInitPrior = 0.562). The “PlotRateThroughTime” function in BAMMtools was used to plot the variation in dynamic rate (speciation, extinction, and net diversification rates) among tree lineages across the section. To better understand diversification rates within different regions, the diversification rates between HMD and non-HMD regions were explored and compared.

### Ancestral niche reconstruction

An occurrence database for sect. *Impatiens* was compiled by integrating specimen records from the Chinese Virtual Herbarium (http://www.cvh.ac.cn/) and Global Biodiversity Information Facility (https://www.gbif.org/), further supplemented with records from our long-term field investigations and from the literature. Duplicate records and errors were removed together with occurrence data outside the native range of the species, as documented in the *Flora of China* [41, 48] and POWO (https://powo.science.kew.org/). Species occurrences were filtered at a resolution of 10 min (~ 18 km at the equator) to correct the collection bias and avoid spatial autocorrelation. This filtering process resulted in the retention of 98,695 occurrence records representing 82 taxa. ArcGIS 10.6 was then used to calculate the species richness pattern based on grid cells with 1°×1° resolution.

The effects of climatic and topography-related environmental variables on geographical patterns in species diversity and niche differentiation were evaluated using multiple linear regression analysis and principal components analysis (PCA). First, data for 20 environment variables (elevation and 19 bioclimatic variables) were downloaded at a spatial resolution of 10 min from WorldClim (http://www.worldclim.org/) and the elevation range within a 10 min × 10 min grid cell was used to represent habitat heterogeneity. Next, the values of 19 bioclimatic variables and the elevation of the 98,695 records were extracted, after which the Pearson correlations among these environmental variables were calculated to check for multicollinearity. After removing the highly correlated variables (Pearson’s *r* > |0.7|) (Table S4), elevation and eight bioclimatic variables (i.e., BIO1: annual mean temperature; BIO2: mean diurnal temperature range; BIO3: isothermality; BIO4: temperature seasonality; BIO8: mean temperature of wettest quarter; BIO9: mean temperature of the driest quarter; BIO12: annual precipitation; BIO14: precipitation during the driest month) were selected for the PCA. Multiple linear regression models of species richness vs. habitat heterogeneity, elevation and climate were then built. The relative weight or relative importance of each variable in explaining the variance of the model was calculated using the “relweights” function.

Based on the results of the multiple linear regression analysis, the annual mean temperature and elevation were selected to analyze ancestral niche reconstruction. For each species, each of these two values was determined as the average of all distribution points of the species. Ancestral states of these two environmental variables were inferred for sect. *Impatiens* using the fastAnc function in R package phytools v. 0.7–00 [106].

### Electronic supplementary material

Below is the link to the electronic supplementary material.

Supplementary Material 1

Supplementary Material 2

## Data Availability

Newly sequenced and other published plastomes in this study can be found in GenBank (https://www.ncbi.nlm.nih.gov/genbank/), and the accession numbers are shown in Table S1.

## List of abbreviations

AICc: Corrected Akaike Information Criterion
BAMM: Bayesian analysis of macro-evolutionary mixtures
BI: Bayesian inference
BS: Bootstrap
ca.: Circa
CDSs: Plastid coding genes
CTAB: Cetyl trimethyl ammonium bromide
DEC: Dispersal-extinction-cladogenesis
DIVA: Dispersal-vicariance
HDM: Hengduan Mountains
HPD: Highest posterior density
Ma: Million years ago
MCC: Maximum clade credibility
MCMC: Markov chain Monte Carlo
ML: Maximum likelihood
non-HDM: non-Hengduan Mountains
PP: Posterior probability
QTP: Qinghai-Tibet Plateau
SD: Standard deviation
