# Supplementary material for: Past climate cooling and orogenesis of the Hengduan Mountains have influenced the evolution of Impatiens sect. Impatiens (Balsaminaceae) in the Northern Hemisphere
Source: BMC Plant Biol. 2023 Nov 29;23:600. doi: 10.1186/s12870-023-04625-w (PMC10685625; doi:10.1186/s12870-023-04625-w)
Supplement: Supplementary file 1 — Supplementary Material 1 [file 12870_2023_4625_MOESM1_ESM.doc]

**Additional file 1**

**Table S1.** Taxa, voucher information, and GenBank accession numbers for the sequences used in this study.

**Table S2.** Comparison of the fit of different models of biogeographical range evolution and model-specific estimates for the different parameters.

**Table S3.** Results of a linear regression of environmental variables with respect to the species richness of *Impatiens* sect. *Impatiens***.**

**Table S4.** Correlation analysis of environment variables

**Table S1.** Taxa, voucher information, and GenBank accession numbers for the sequences used in this study.

| Species | Locality | Voucher | GenBankaccession |
| --- | --- | --- | --- |
| *Impatiens alpicola* Y. L. Chen et Y. Q. Lu | Sichuan, China | XXX | NC_053940 |
| *Impatiens davidii* Franchet | — | — | NC_058801 |
| *Impatiens fanjingshanica* Y. L. Chen | Guizhou, China | SWFU-IBFJS20171030 | NC_059944 |
| *Impatiens macrovexilla* var. *yaoshanensis* 2 S. X. Yu et al. | — | — | OK310516 |
| *Impatiens macrovexilla* Y. L. Chen | — | — | OK310515 |
| *Impatiens piufanensis* J. D. Hooker | — | — | NC_037401 |
| *Impatiens balsamina* 2 L. | — | — | NC_059942 |
| *Impatiens chlorosepala* Hand.-Mazz. | Guangxi, China | SWFU-IBLE20161008 | NC_059943 |
| *Impatiens glandulifera* Royle | Berkshire, England | HB10 | NC_044718 |
| *Impatiens hawkeri* W. Bull | — | — | NC_048520 |
| *Impatiens loulanensis* Hook. f. | Guizhou, China | SWFU-IBLN20161013 | NC_059947 |
| *Impatiens monticola* Hook. f. | Guizhou, China | SWFU-IBSD20180823 | NC_058205 |
| *Impatiens pritzelii* Hook. f. | Chongqing, China | Q Wang JYS20190601 (SWCTU) | NC_047191 |
| *Impatiens stenosepala* Pritz. ex Diels | Guizhou, China | SWFU-IBZE20171030 | NC_059948 |
| *Impatiens uliginosa* Franch. | Yunnan, China | SWFU-IBDSJF20180810 | NC_059760 |
| *Impatiens walleriana* J. D. Hooker | — | — | NC_059949 |
| *Hydrocera triflora* (L.) Wight. et Arn. | Hainan, China | HIB-lzz18 (HIB) | NC_037400 |
| *Marcgravia coriacea* Vahl | — | 19672994 (KUN) | NC_041255 |
| *Impatiens apsotis* Hook. f. | Sichuan, China | SX Yu et al. WS-419 (PE) | OR135418 |
| *Impatiens atrosanguinea* (Nakai) B.U. Oh & W.P. Hong | Hwangmaesan, Korea | — 2041 (PE) | OR135508 |
| *Impatiens baishaensis* B. Ding & H. P. Deng | Sichuan, China | TT Xue et al. Y12574 (PE) | OR135410 |
| *Impatiens barbata* Comber | Yunnan, China | H Peng et al. 6665 (PE) | OR135475 |
| *Impatiens bijieensis* X.X.Bai & L.Y.Ren | Guizhou, China | XX Bai et al. DJCP 20210820 (PE) | OR135434 |
| *Impatiens bodinieri* Hook. f. | Guangxi, China | SX Yu 3715 (PE) | OR135496 |
| *Impatiens capensis* Meerb. | North Carolina, America | James S.Miller et al. 8923 (PE) | OR135466 |
| *Impatiens chekiangensis* Y. L. Chen | Zhejiang, China | CF Zhang 1858 (PE) | OR135511 |
| *Impatiens chiulungensis* Y. L. Chen | Sichuan, China | SX Yu 3989 (PE) | OR135488 |
| *Impatiens commellinoides* Hand.-Mazz. | Jiangxi, China | Southern Jiangxi Expedition Team 473 (PE) | OR135517 |
| *Impatiens compta* 1 Hook. f. | Chongqing, China | B Ding BD201509041 (PE) | OR135438 |
| *Impatiens compta* 2 Hook. f. | Hubei, China | SX Yu et al. 8339 (PE) | OR135470 |
| *Impatiens conaensis* Y. L. Chen | Xizang, China | FLPH 12-0660 (PE) | OR135520 |
| *Impatiens corchorifolia* Franch. | Yunnan, China | SX Yu et al. 6403 (PE) | OR135477 |
| *Impatiens cornucopia* 1 Franch. | Sichuan, China | SX Yu et al. 9858 (PE) | OR135464 |
| *Impatiens* *cornucopia* 2 Franch. | Yunnan, China | SX Yu 7 (PE) | OR135521 |
| *Impatiens cornutisepala* S. X. Yu | Guangxi, China | SX Yu 4023 (PE) | OR135487 |
| *Impatiens delavayi* Franch. | Yunnan, China | Q Fan 19108 (PE) | OR135459 |
| *Impatiens dicentra* 1 Franch. ex Hook. f. | Guizhou, China | SX Yu et al. 8586 (PE) | OR135467 |
| *Impatiens dicentra* 2 Franch. ex Hook. f. | Chongqing, China | B Ding BD201508032 (PE) | OR135441 |
| *Impatiens epilobioides* Y. L. Chen | Sichuan, China | WB Xu et al. Y12168 (PE) | OR135416 |
| *Impatiens faberi* Hook. f. | Sichuan, China | SX Yu 4087 (PE) | OR135485 |
| *Impatiens fargesii* Hook. f. | Chongqing, China | B Ding BD201509026 (PE) | OR135439 |
| *Impatiens fenghwaiana* Y. L. Chen | Jiangxi, China | Lushan Botanical Garden Expedition Team WNB2004-00100 (PE) | OR135420 |
| *Impatiens fissicornis* Maxim. | Shaanxi, China | — I 0388 (PE) | OR135431 |
| *Impatiens forrestii* Hook. f. | Yunnan, China | Yunnan Expedition Team YN-ET 1563 (PE) | OR135407 |
| *Impatiens* *furcillata* Hemsl. | Gangwon-do, Korea | CH Nam, et al. N120191 (PE) | OR135429 |
| *Impatiens ganpiuana* Hook. f. | Guizhou, China | — 2107 (PE) | OR135507 |
| *Impatiens guiqingensis* S. X. Yu | Gansu, China | CF Zhang 1275 (PE) | OR135514 |
| *Impatiens henanensis* Y. L. Chen | Henan, China | Americans (s. n.) — (PE) | OR135445 |
| *Impatiens henryi* Pritz. ex Diels | Hubei, China | SX Yu et al. 8169 (PE) | OR135471 |
| *Impatiens huangyanensis* X. F. Jin & B. Y. Ding | Zhejiang, China | SX Yu et al. ZJ386 (PE) | OR135404 |
| *Impatiens imbecilla* Hook. f. | Sichuan, China | B Ding BD201509070 (PE) | OR135437 |
| *Impatiens lacinulifera* Y. L. Chen | Gansu, China | Baishuijiang Expedition Team 1886 (PE) | OR135510 |
| *Impatiens latebracteata* Hook. f. | Shaanxi, China | HN Qin et al. 19320 (PE) | OR135458 |
| *Impatiens lateristachys* Y. L. Chen et Y. Q. Lu | Sichuan, China | WB Xu et al. Y12231 (PE) | OR135414 |
| *Impatiens lecomtei* Hook. f. | Xizang, China | XH Jin et al. DLJ-ET 3303 (PE) | OR135432 |
| *Impatiens leptocaulon* Hook. f. | Guangxi, China | SX Yu 3684 (PE) | OR135498 |
| *Impatiens liupanshuiensis* X.X.Bai & T.H.Yuan | Guizhou, China | XX Bai et al. BXX 368 (PE) | OR135436 |
| *Impatiens lixianensis* S. X. Yu | Sichuan, China | SX Yu 3922 (PE) | OR135489 |
| *Impatiens longialata* 1 E. Pritz. ex Diels | Chongqing, China | SX Yu et al. 8479 (PE) | OR135468 |
| *Impatiens longialata* 2 E. Pritz. ex Diels | Chongqing, China | B Ding BD201509021 (PE) | OR135440 |
| *Impatiens lucorum* Hook. f. | Sichuan, China | WB Xu et al. Y12223 (PE) | OR135415 |
| *Impatiens macrovexilla* var. *yaoshanensis* 1 S. X. Yu et al. | Guizhou, China | YB Luo 516 (PE) | OR135516 |
| *Impatiens menghuochengensis* Q Luo. | Sichuan, China | Q Luo 131015 (PE) | OR135449 |
| *Impatiens microstachys* Hook. f. | Sichuan, China | TT Xue Y12565 (PE) | OR135411 |
| *Impatiens nasuta* Hook. f. | Chongqing, China | B Ding BD201508012 (PE) | OR135443 |
| *Impatiens neglecta* Y. L. Xu et Y. L. Chen | Anhui, China | HN Qin et al. 19942A (PE) | OR135457 |
| *Impatiens noli-tangere* L. | Zhejiang, China | HN Qin et al. 20090 (PE) | OR135455 |
| *Impatiens notolopha* Maximowicz | Sichuan, China | DE Boufford et al. 44521 (PE) | OR135451 |
| *Impatiens nubigena* W. W. Smith | Yunnan, China | WB Xu et al. QTP882 (PE) | OR135425 |
| *Impatiens oxyanthera* J. D. Hooker | Sichuan, China | SX Yu 3269 (PE) | OR135500 |
| *Impatiens pallida* Nutt. | Tennessee, America | LR Phillippe et al. 42222 (PE) | OR135453 |
| *Impatiens paradoxa* C. S. Chu et H. W. Yang | Henan, China | CS Zhu et al. 920312 (PE) | OR135448 |
| *Impatiens platychlaena* Hook. f. | Sichuan, China | SX Yu 3783 (PE) | OR135491 |
| *Impatiens plicatisepala* C.Y.Zou, Yan Liu & S.X.Yu | Guangxi, China | SX Yu 4032 (PE) | OR135486 |
| *Impatiens potaninii* Maxim. | Chongqing, China | B Ding BD201508015 (PE) | OR135442 |
| *Impatiens pterosepala* Hook. f. | Hubei, China | SX Yu et al. 8400 (PE) | OR135469 |
| *Impatiens quadriloba* K. M. Liu & Y. L. Xiang | Sichuan, China | DE Boufford et al. 44474 (PE) | OR135452 |
| *Impatiens rectirostrata* Y. L. Chen | Sichuan, China | SX Yu 3791 (PE) | OR135490 |
| *Impatiens reptans* Hook. f. | Hunan, China | SX Yu et al. M815 (PE) | OR135430 |
| *Impatiens rhombifolia* Y. Q. Lu et Y. L. Chen | Guangxi, China | SX Yu 3709 (PE) | OR135497 |
| *Impatiens shennongensis* Q. Wang & H. P. Deng | Hubei, China | SX Yu et al. 8149 (PE) | OR135472 |
| *Impatiens soulieana* Hook. f. | Sichuan, China | SX Yu et al. WS-346 (PE) | OR135419 |
| *Impatiens subecalcarata* (Hand.-Mazz.) Y. L. Chen | Yunnan, China | Southeast Tibet Expedition Team SET_ET 70 (PE) | OR135424 |
| *Impatiens suichangensis* Y. L. Xu et Y. L. Chen | Zhejiang, China | SX Yu et al. ZJ374 (PE) | OR135405 |
| *Impatiens sutchuenensis* 1 Franchet ex J. D. Hooker | Chongqing, China | B Ding BD201508006 (PE) | OR135444 |
| *Impatiens sutchuenensis* 2 Franchet ex J. D. Hooker | Chongqing, China | B Ding BD201509010 (PE) | OR135435 |
| *Impatiens tayemonii* Hayata | Taiwan, China | B Bartholomew et al. 14637 (PE) | OR135461 |
| *Impatiens textorii* Miquel | Gyeongsangnam, Korea | YH Cho et al. NAM-08015-058 (PE) | OR135428 |
| *Impatiens tienchuanensis* Y. L. Chen | Sichuan, China | SX Yu et al. WS-761 (PE) | OR135417 |
| *Impatiens tienmushanica* var. *longicalcarata* Y.L. Xu et Y.L. Chen | Zhejiang, China | SX Yu et al. ZJ387 (PE) | OR135403 |
| *Impatiens tienmushanica* Y. L. Chen | Zhejiang, China | HN Qin et al. 20089 (PE) | OR135456 |
| *Impatiens tortisepala* Hook. f. | Sichuan, China | WB Xu et al. Y12306 (PE) | OR135412 |
| *Impatiens undulata* Y. L. Chen et Y. Q. Lu | Sichuan, China | Liden & Wang 2005-3 (PE) | OR135509 |
| *Impatiens uniflora* Hayata | Taiwan, China | JR Chen 97504 (PE) | OR135450 |
| *Impatiens vittata* Franch. | Sichuan, China | WB Xu et al. Y12266 (PE) | OR135413 |
| *Impatiens wuyuanensis* Y. L. Chen | Jiangxi, China | XD Yang Y15156 (PE) | OR135408 |
| *Impatiens xanthocephala* W. W. Smith | Sichuan, China | SX Yu et al. 9962 (PE) | OR135462 |
| *Impatiens yunlingensis* S.X. Yu, Chang Y. Xia & J.H. Yu | Sichuan, China | SX Yu et al. 9881 (PE) | OR135463 |
| *I. sp* 1 | Jiangxi, China | XD Yang Y15124 (PE) | OR135409 |
| *I. sp* 2 | Sichuan, China | DE Boufford et al. 27887 (PE) | OR135454 |
| *Impatiens angulata* S. X. Yu | Guangxi, China | Zhongzhilian-Guangxi Expedition 3746 (PE) | OR135494 |
| *Impatiens apalophylla* Hook. f. | Guangxi, China | Zhongzhilian-Guangxi Expedition 4324 (PE) | OR135483 |
| *Impatiens arguta* 1 Hook. f. et Thoms. | Yunnan, China | SX Yu et al. 6348 (PE) | OR135478 |
| *Impatiens arguta* 2 Hook. f. et Thoms. | Yunnan, China | CF Zhang 1530 (PE) | OR135512 |
| *Impatiens arguta* 3 Hook. f. et Thoms. | Xizang, China | SX Yu et al. 5406 (PE) | OR135482 |
| *Impatiens aureliana* Hook. f. | Kachin, Myanmar | XH Jin et al. PT-ET157 (PE) | OR135427 |
| *Impatiens balsamina* 1 L. | Hunan, China | HF Wang 84 (PE) | OR135518 |
| *Impatiens burtonii* Hook.f. | — | YJ Guo 15CS10515 (PE) | OR135519 |
| *Impatiens chinensis* L. | Guangxi, China | Zhongzhilian-Guangxi Expedition 2794 (PE) | OR135505 |
| *Impatiens clavigera* J. D. Hooker | Guangxi, China | SX Yu et al. 450603171206033LY (PE) | OR135446 |
| *Impatiens cymbifera* Hook. f. | Xizang, China | YS Chen et al. 706 (PE) | OR135515 |
| *Impatiens duclouxii* Hook. f. | Guangxi, China | Zhongzhilian-Guangxi Expedition 3098 (PE) | OR135503 |
| *Impatiens gongshanensis* Y. L. Chen | Yunnan, China | XH Jin et al. ST0789 (PE) | OR135422 |
| *Impatiens hunanensis* Y. L. Chen | Guangxi, China | SX Yu 3651 (PE) | OR135499 |
| *Impatiens kamerunensis* Warb. | Bioco, Equatorial Guinea | Carvalho 2778 (PE) | OR135506 |
| *Impatiens longlinensis* S.X.Yu | Guangxi, China | SX Yu 8087 (PE) | OR135474 |
| *Impatiens mengtszeana* J. D. Hooker | Yunnan, China | SX Yu et al. 6314 (PE) | OR135479 |
| *Impatiens morsei* Hook. f. | Guangxi, China | Zhongzhilian-Guangxi Expedition 1394 (PE) | OR135513 |
| *Impatiens nyimana* Marq. et Airy-Shaw | Xizang, China | SX Yu et al. 6043 (PE) | OR135481 |
| *Impatiens pandurata* Y.H. Tan & S.X. Yu | Yunnan, China | SX Yu 8972DQG (PE) | OR135465 |
| *Impatiens parvisepala* S. X. Yu & Y. T. Hou | Guangxi, China | SX Yu 3754 (PE) | OR135493 |
| *Impatiens pingxiangensis* 1 H. Y. Bi & S. X. Yu | Guangxi, China | YF Chen et al. 08131 (PE) | OR135473 |
| *Impatiens pingxiangensis* 2 H. Y. Bi & S. X. Yu | Guangxi, China | SX Yu 3088 (PE) | OR135504 |
| *Impatiens puberula* DC. | Sagarmatha, Nepal | M Wakabayashi et al. 9715298 (PE) | OR135447 |
| *Impatiens radiata* Hook. f. | Yunnan, China | XH Jin et al. ST2074 (PE) | OR135421 |
| *Impatiens rubrostriata* J. D. Hooker | Yunnan, China | SX Yu 4208 (PE) | OR135484 |
| *Impatiens serrata* Benth. ex Hook. f. et Thoms. | Xizang, China | L Wei et al. 15410 (PE) | OR135460 |
| *Impatiens siculifer* Hook. f. | Yunnan, China | SX Yu et al. 6293 (PE) | OR135480 |
| *Impatiens stenantha* Hook. f. | Kachin, Myanmar | XH Jin et al. PT-ET1113 (PE) | OR135426 |
| *Impatiens taronensis* Hand.-Mazz. | Yunnan, China | SX Yu et al. 6429 (PE) | OR135476 |
| *Impatiens tianlinensis* S.X. Yu & L.J. Zhang | Guangxi, China | SX Yu 3731 (PE) | OR135495 |
| *Impatiens tubulosa* Hemsl. | Guangxi, China | SX Yu 3772 (PE) | OR135492 |
| *Impatiens unguiculata* 1 K.M .Liu & Y.Y. Cong | Guangxi, China | SX Yu YSX0545 (PE) | OR135406 |
| *Impatiens unguiculata* 2 K.M. Liu & Y.Y. Cong | Guangxi, China | SX Yu 3220 (PE) | OR135501 |
| *Impatiens xanthina* Comber | Yunnan, China | XH Jin et al. ST0151 (PE) | OR135423 |
| *Impatiens yui* S.H.Huang | Yunnan, China | XH Jin et al. DLJ-ET 2092 (PE) | OR135433 |
| *I. sp* 3 | Guangxi, China | SX Yu 3128 (PE) | OR135502 |

**Table S2.** Comparison of the fit of different models of biogeographical range evolution and model-specific estimates for the different parameters. Ln*L*, log-likelihood; *d*, rate of range expansion; *e*, rate of range contraction; *j*, relative per-event weight of jump dispersal; AICc, corrected Akaike Information Criterion; AICc_wt, weighted AICc. The best model with the lowest AICc vaule is in bold.

| Models | Ln*L* | Numparams | *d* | *e* | *j* | AICc | AICc_wt |
| --- | --- | --- | --- | --- | --- | --- | --- |
| DEC | -99.11 | 2 | 0.011 | 1.0e-12 | 0 | 202.4 | 0.0013 |
| **DEC+J** | **-91.37** | **3** | **0.0044** | **1.0e-12** | **0.028** | **189.1** | **1.00** |
| DIVALIKE | -112.2 | 2 | 0.019 | 4.0 e-9 | 0 | 228.6 | 2.6e-9 |
| DIVALIKE+J | -98.77 | 3 | 0.0058 | 1.0e-12 | 0.032 | 203.9 | 0.0006 |
| BAYAREALIKE | -141.8 | 2 | 0.0064 | 0.12 | 0 | 287.8 | 3.6e-22 |
| BAYAREALIKE+J | -103.1 | 3 | 0.0034 | 1.0 e-7 | 0.039 | 212.4 | 8.5e-6 |

**Table S3. Results of a linear regression of environmental variables with respect to the species richness of *Impatiens* sect. *Impatiens*.**

|  | Estimate | Std. error | t value | Pr(>|t|) | Relative importance (%) |
| --- | --- | --- | --- | --- | --- |
| (Intercept) | 1.4090 | 0.4977 | 2.832 | 0.00468* | – |
| Elevation range | 0.0005 | 0.00007 | 7.733 | 1.68×10^–14^** | 22.40 |
| BIO1 | 0.1383 | 0.0143 | 9.671 | <2.2×10^–16^** | 18.32 |
| BIO2 | –0.0282 | 0.0396 | –0.714 | 0.47549 | 0.99 |
| BIO3 | –0.0647 | 0.0162 | –3.986 | 6.96×10^–5^** | 3.72 |
| BIO4 | 0.0006 | 0.0004 | 1.343 | 0.17928 | 3.64 |
| BIO8 | 0.0026 | 0.0079 | 0.330 | 0.74153 | 12.24 |
| BIO9 | –0.0112 | 0.0068 | –1.635 | 0.10215 | 5.91 |
| BIO12 | 0.0004 | 0.0001 | 3.097 | 0.00198* | 10.40 |
| BIO14 | –0.0058 | 0.0022 | –2.673 | 0.00758* | 5.68 |
| Elevation | 0.0006 | 0.00007 | 9.319 | <2.2×10^–16^** | 16.70 |

Adjusted R–squared=0.306; p–value < 2.2×10^–16^; * p < 0.05; **p < 0.01; ***p < 0.001.

**Table S4. Correlation analysis of environment variables.**

|  | Elevation | IOBIO1 | BIOIO2 | BIOIO3 | IOBIO4 | BIO5 | BIO6 | BIO7 | BIO8 | BIO9 | BIO10 | BIO11 | BIO12 | BIO13 | BIO14 | BIO15 | BIO16 | BIO17 | BIO18 | BIO19 |
| --- | --- | --- | --- | --- | --- | --- | --- | --- | --- | --- | --- | --- | --- | --- | --- | --- | --- | --- | --- | --- |
| Elevation | 1.00 | -0.11 | 0.11 | 0.33 | -0.19 | -0.30 | -0.05 | -0.12 | -0.04 | -0.10 | -0.27 | 0.00 | 0.10 | 0.29 | -0.21 | 0.47 | 0.29 | -0.21 | 0.34 | -0.19 |
| BIO1 | -0.11 | 1.00 | 0.13 | 0.56 | -0.45 | 0.71 | 0.79 | -0.35 | 0.32 | 0.69 | 0.82 | 0.89 | 0.50 | 0.41 | 0.34 | -0.13 | 0.42 | 0.37 | 0.36 | 0.29 |
| BIO2 | 0.11 | 0.13 | 1.00 | 0.37 | 0.52 | 0.62 | -0.38 | 0.70 | 0.27 | -0.13 | 0.46 | -0.18 | 0.04 | -0.02 | 0.10 | 0.07 | -0.03 | 0.11 | 0.04 | -0.02 |
| BIO3 | 0.33 | 0.56 | 0.37 | 1.00 | -0.58 | 0.23 | 0.56 | -0.39 | -0.03 | 0.55 | 0.23 | 0.65 | 0.26 | 0.09 | 0.35 | -0.20 | 0.10 | 0.36 | 0.08 | 0.31 |
| BIO4 | -0.19 | -0.45 | 0.52 | -0.58 | 1.00 | 0.28 | -0.87 | 0.97 | 0.27 | -0.66 | 0.14 | -0.80 | -0.25 | -0.12 | -0.29 | 0.29 | -0.14 | -0.29 | -0.05 | -0.34 |
| BIO5 | -0.30 | 0.71 | 0.62 | 0.23 | 0.28 | 1.00 | 0.17 | 0.39 | 0.52 | 0.25 | 0.97 | 0.34 | 0.30 | 0.24 | 0.20 | -0.03 | 0.24 | 0.23 | 0.25 | 0.10 |
| BIO6 | -0.05 | 0.79 | -0.38 | 0.56 | -0.87 | 0.17 | 1.00 | -0.84 | -0.00 | 0.78 | 0.33 | 0.97 | 0.39 | 0.25 | 0.35 | -0.30 | 0.27 | 0.36 | 0.18 | 0.37 |
| BIO7 | -0.12 | -0.35 | 0.70 | -0.39 | 0.97 | 0.39 | -0.84 | 1.00 | 0.28 | -0.59 | 0.23 | -0.72 | -0.19 | -0.10 | -0.21 | 0.26 | -0.12 | -0.21 | -0.03 | -0.29 |
| BIO8 | -0.04 | 0.32 | 0.27 | -0.03 | 0.27 | 0.52 | -0.00 | 0.28 | 1.00 | -0.26 | 0.52 | 0.07 | -0.08 | 0.15 | -0.34 | 0.33 | 0.13 | -0.33 | 0.31 | -0.46 |
| BIO9 | -0.10 | 0.69 | -0.13 | 0.55 | -0.66 | 0.25 | 0.78 | -0.59 | -0.26 | 1.00 | 0.35 | 0.80 | 0.49 | 0.24 | 0.55 | -0.37 | 0.27 | 0.56 | 0.09 | 0.62 |
| BIO10 | -0.27 | 0.82 | 0.46 | 0.23 | 0.14 | 0.97 | 0.33 | 0.23 | 0.52 | 0.35 | 1.00 | 0.48 | 0.38 | 0.36 | 0.20 | 0.02 | 0.35 | 0.23 | 0.35 | 0.11 |
| BIO11 | 0.00 | 0.89 | -0.18 | 0.65 | -0.80 | 0.34 | 0.97 | -0.72 | 0.07 | 0.80 | 0.48 | 1.00 | 0.46 | 0.33 | 0.38 | -0.25 | 0.34 | 0.40 | 0.26 | 0.38 |
| BIO12 | 0.10 | 0.50 | 0.04 | 0.26 | -0.25 | 0.30 | 0.39 | -0.19 | -0.08 | 0.49 | 0.38 | 0.46 | 1.00 | 0.79 | 0.69 | -0.16 | 0.84 | 0.72 | 0.71 | 0.72 |
| BIO13 | 0.29 | 0.41 | -0.02 | 0.09 | -0.12 | 0.24 | 0.25 | -0.10 | 0.15 | 0.24 | 0.36 | 0.33 | 0.79 | 1.00 | 0.16 | 0.42 | 0.99 | 0.19 | 0.93 | 0.25 |
| BIO14 | -0.21 | 0.34 | 0.10 | 0.35 | -0.29 | 0.20 | 0.35 | -0.21 | -0.34 | 0.55 | 0.20 | 0.38 | 0.69 | 0.16 | 1.00 | -0.72 | 0.22 | 0.99 | 0.10 | 0.92 |
| BIO15 | 0.47 | -0.13 | 0.07 | -0.20 | 0.29 | -0.03 | -0.30 | 0.26 | 0.33 | -0.37 | 0.02 | -0.25 | -0.16 | 0.42 | -0.72 | 1.00 | 0.37 | -0.71 | 0.44 | -0.62 |
| BIO16 | 0.29 | 0.42 | -0.03 | 0.10 | -0.14 | 0.24 | 0.27 | -0.12 | 0.13 | 0.27 | 0.35 | 0.34 | 0.84 | 0.99 | 0.22 | 0.37 | 1.00 | 0.25 | 0.93 | 0.30 |
| BIO17 | -0.21 | 0.37 | 0.11 | 0.36 | -0.29 | 0.23 | 0.36 | -0.21 | -0.33 | 0.56 | 0.23 | 0.40 | 0.72 | 0.19 | 0.99 | -0.71 | 0.25 | 1.00 | 0.12 | 0.93 |
| BIO18 | 0.34 | 0.36 | 0.04 | 0.08 | -0.05 | 0.25 | 0.18 | -0.03 | 0.31 | 0.09 | 0.35 | 0.26 | 0.71 | 0.93 | 0.10 | 0.44 | 0.93 | 0.12 | 1.00 | 0.08 |
| BIO19 | -0.19 | 0.29 | -0.02 | 0.31 | -0.34 | 0.10 | 0.37 | -0.29 | -0.46 | 0.62 | 0.11 | 0.38 | 0.72 | 0.25 | 0.92 | -0.62 | 0.30 | 0.93 | 0.08 | 1.00 |
