# Supplementary material for: Past climate cooling and orogenesis of the Hengduan Mountains have influenced the evolution of Impatiens sect. Impatiens (Balsaminaceae) in the Northern Hemisphere
Source: BMC Plant Biol. 2023 Nov 29;23:600. doi: 10.1186/s12870-023-04625-w (PMC10685625; doi:10.1186/s12870-023-04625-w)
Supplement: Supplementary file 2 — Supplementary Material 2 [file 12870_2023_4625_MOESM2_ESM.docx]

**Additional file 2**

**Fig. S1.** Maximum likelihood tree of *Impatiens* sect. *Impatiens* inferred from concatenated nucleotide sequences of 80 plastid coding genes. Numbers above the branches are the bootstrap values.

**Fig. S2.** Bayesian inference tree of *Impatiens* sect. *Impatiens* inferred from concatenated nucleotide sequences of 80 plastid coding genes. Numbers above the branches are the bootstrap values.

**Fig. S3.** ML tree of *Impatiens* sect. *Impatiens* obtained from IQ-TREE. Numbers above the branches are the SH-aLRT test value (SH-aLRT) and the ultrafast bootstrap value (UFboot).

**Fig. S4.** Chronogram of *Impatiens*. Numbers above the branches are the divergence time. Blue bars indicate the 95% highest posterior density intervals. Q.=Quatemary, P.=Pliocene.

**Fig. S5.** Lineage-through-time plot for *Impatiens* sect. *Impatiens*.

**Fig. S6.** Diversification rates of clades of *Impatiens* sect. *Impatiens*. (A) Clade I; (B) Clade II; (C) Clade III; (D) Clade IV; (E) Clade V; (F) Clade VI; (G) Clade VII.

**Fig. S7.** Distribution pattern of the species richness of *Impatiens* sect. *Impatiens*.

**Fig. S8.** Principal component analysis of the environmental factors relevant to *Impatiens* sect. *Impatiens*.

**Fig. S9.** Gravel diagram of principal component analysis.

**Fig. S10.** Five geographical regions of *Impatiens* sect. *Impatiens* (A) Northwest China; (B) Hengduan Mountains; (C) Southeast China; (D) temperate Eurasia; (E) North America.

**
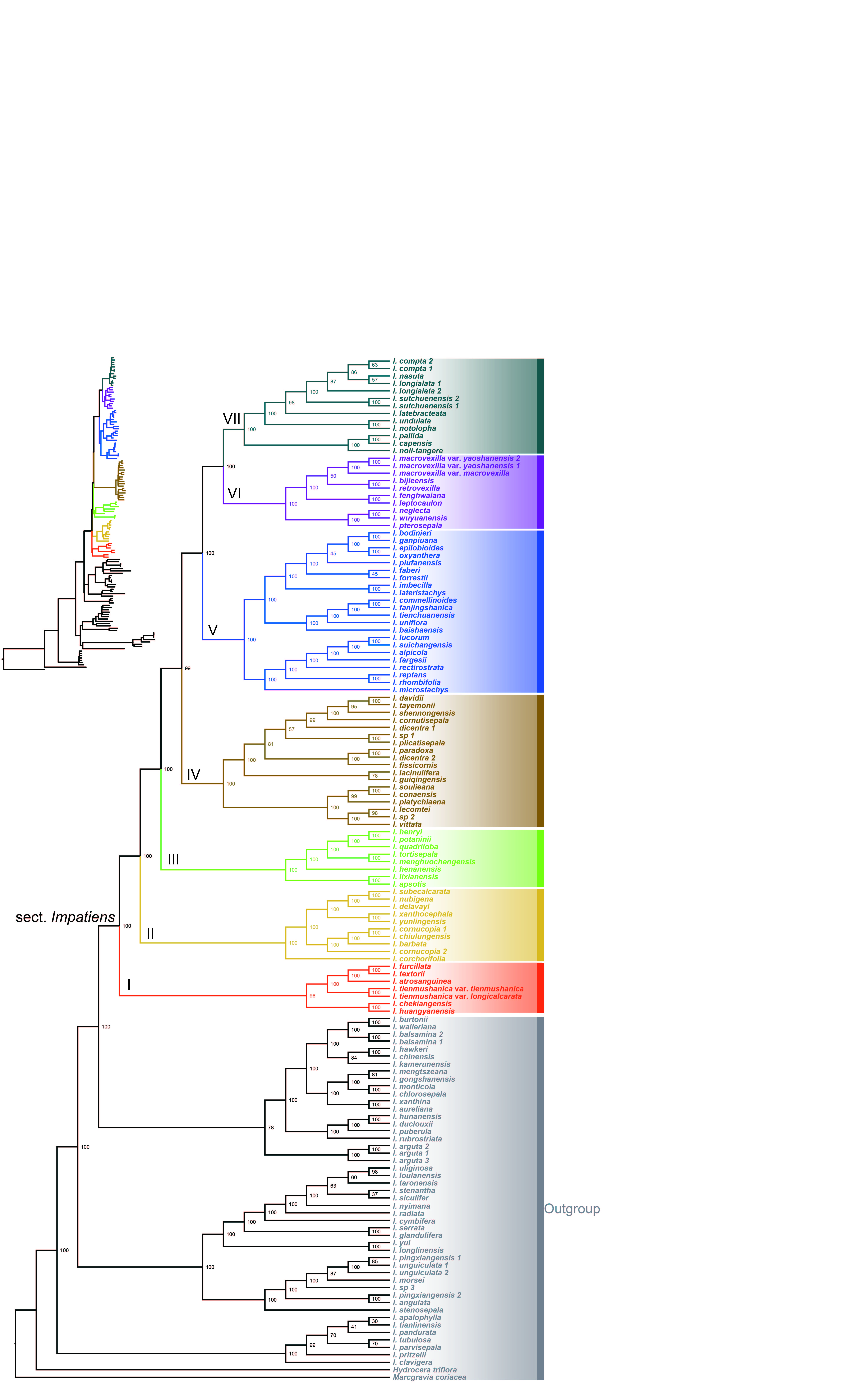
**

**Fig. S1.** Maximum likelihood tree of *Impatiens* sect. *Impatiens* inferred from concatenated nucleotide sequences of 80 plastid coding genes. Numbers above the branches are the bootstrap values.

**
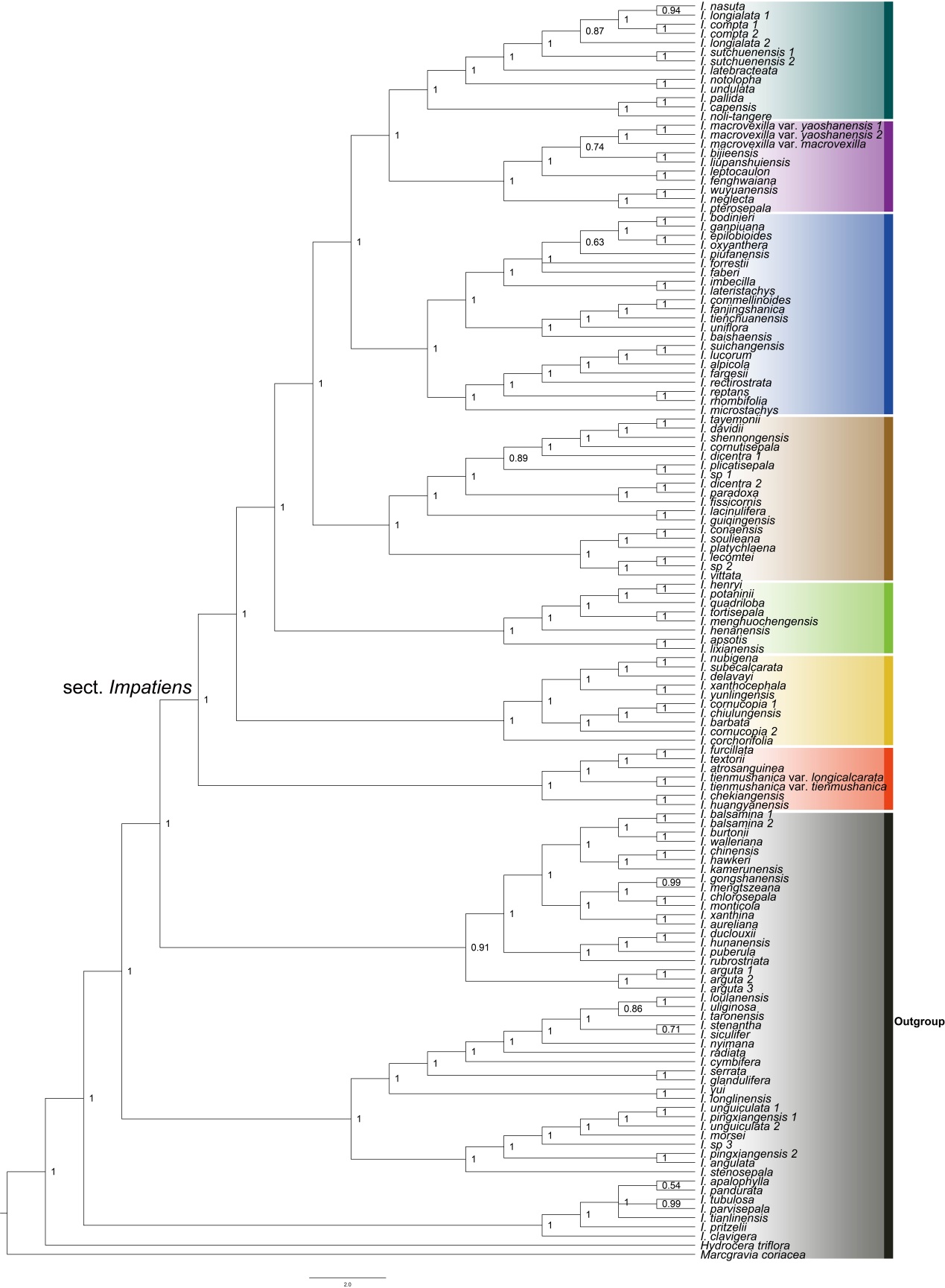
**

**Fig. S2.** Bayesian inference tree of *Impatiens* sect. *Impatiens* inferred from concatenated nucleotide sequences of 80 plastid coding genes. Numbers above the branches are the bootstrap values.

**
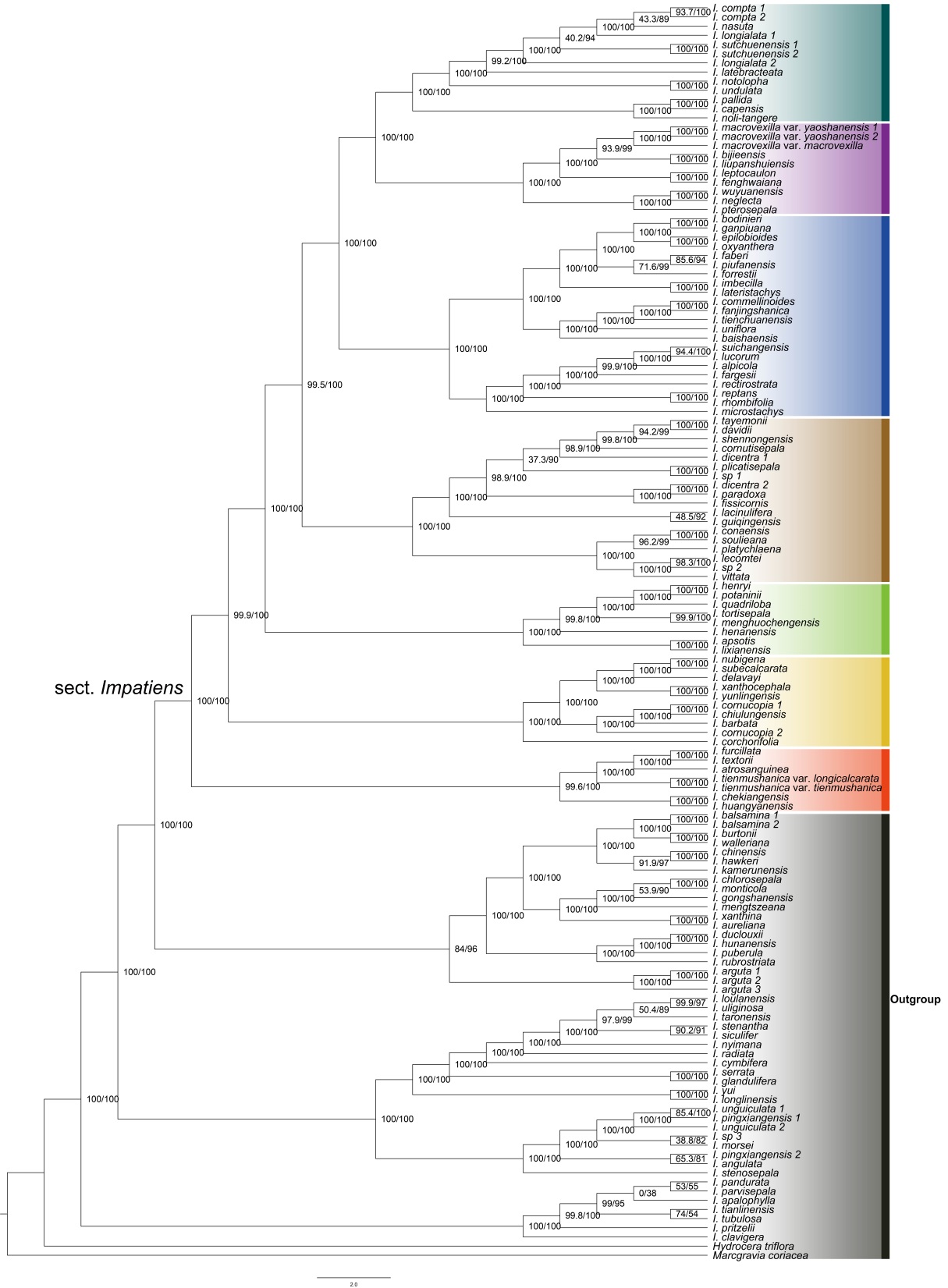
**

**Fig. S3.** ML tree of *Impatiens* sect. *Impatiens* obtained from IQ-TREE. Numbers above the branches are the SH-aLRT test value (SH-aLRT) and the ultrafast bootstrap value (UFboot).

**
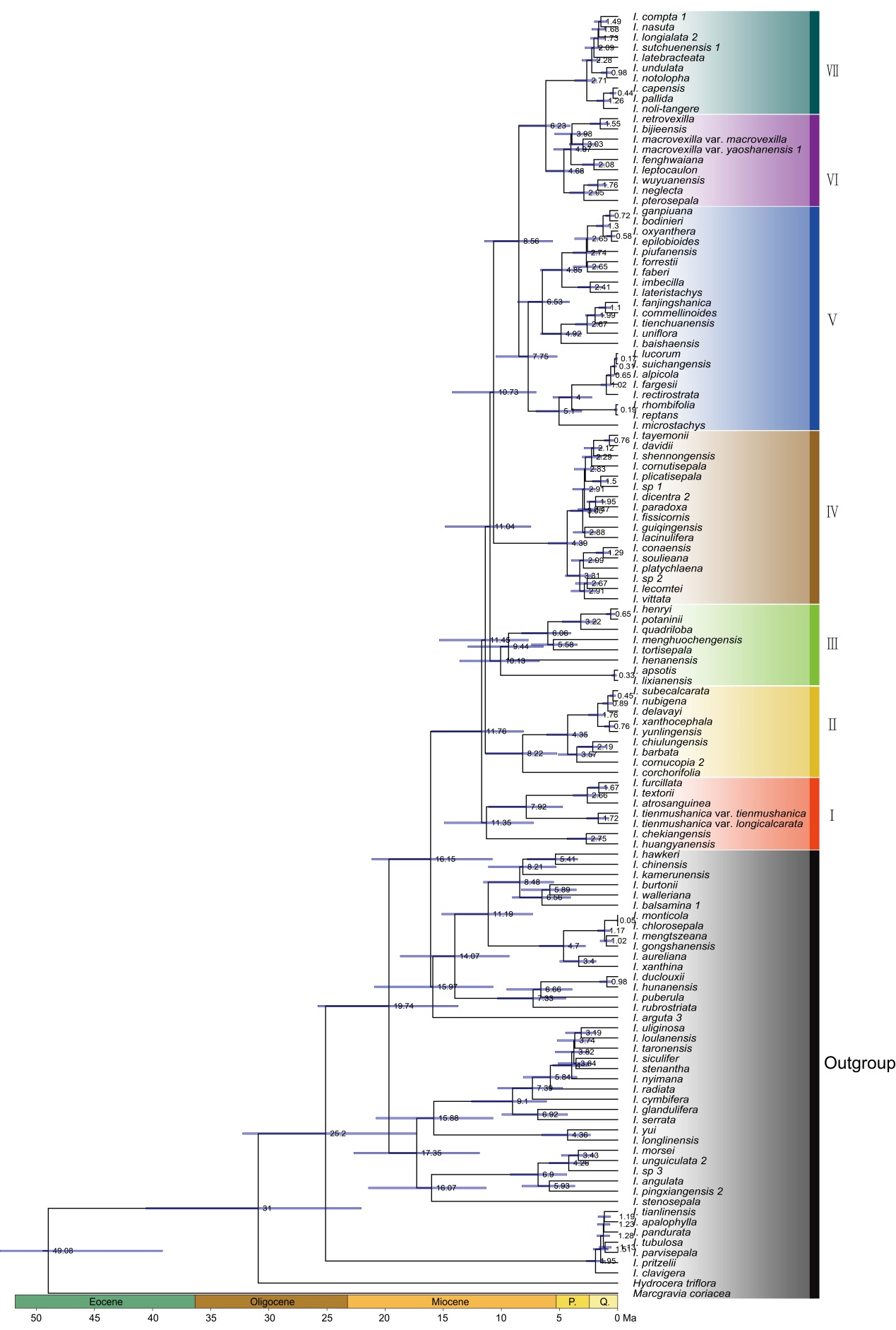
**

**Fig. S4.** Chronogram of *Impatiens*. Numbers above the branches are the divergence time. Blue bars indicate the 95% highest posterior density intervals. Q.=Quatemary, P.=Pliocene.

**
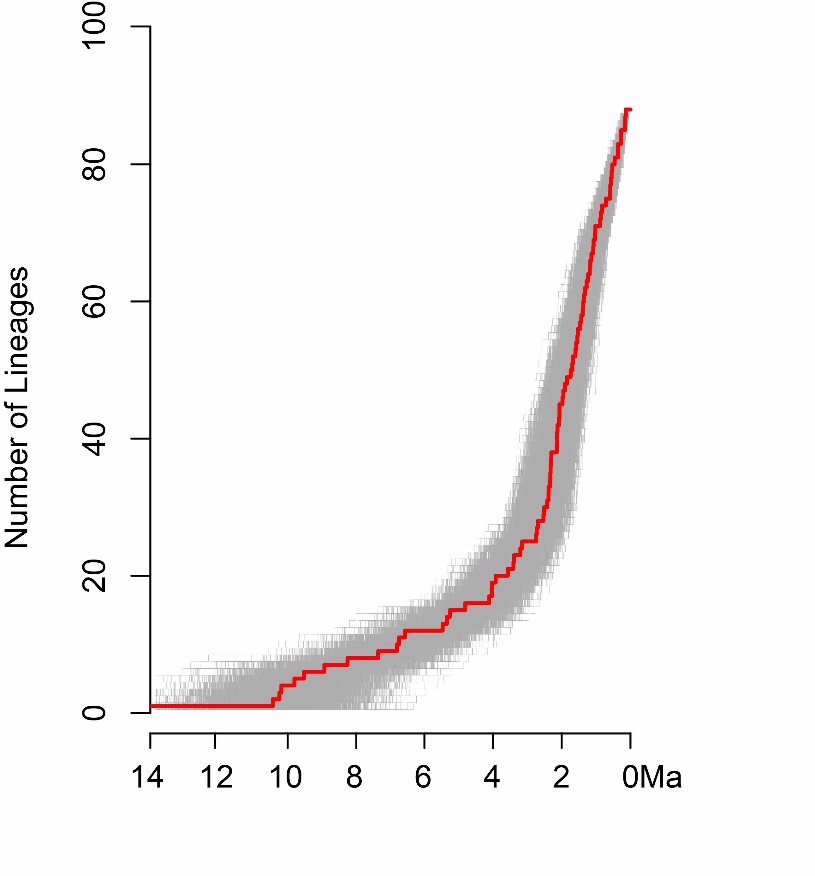
**

**Fig. S5.** Lineage-through-time plot for *Impatiens* sect. *Impatiens*.

**
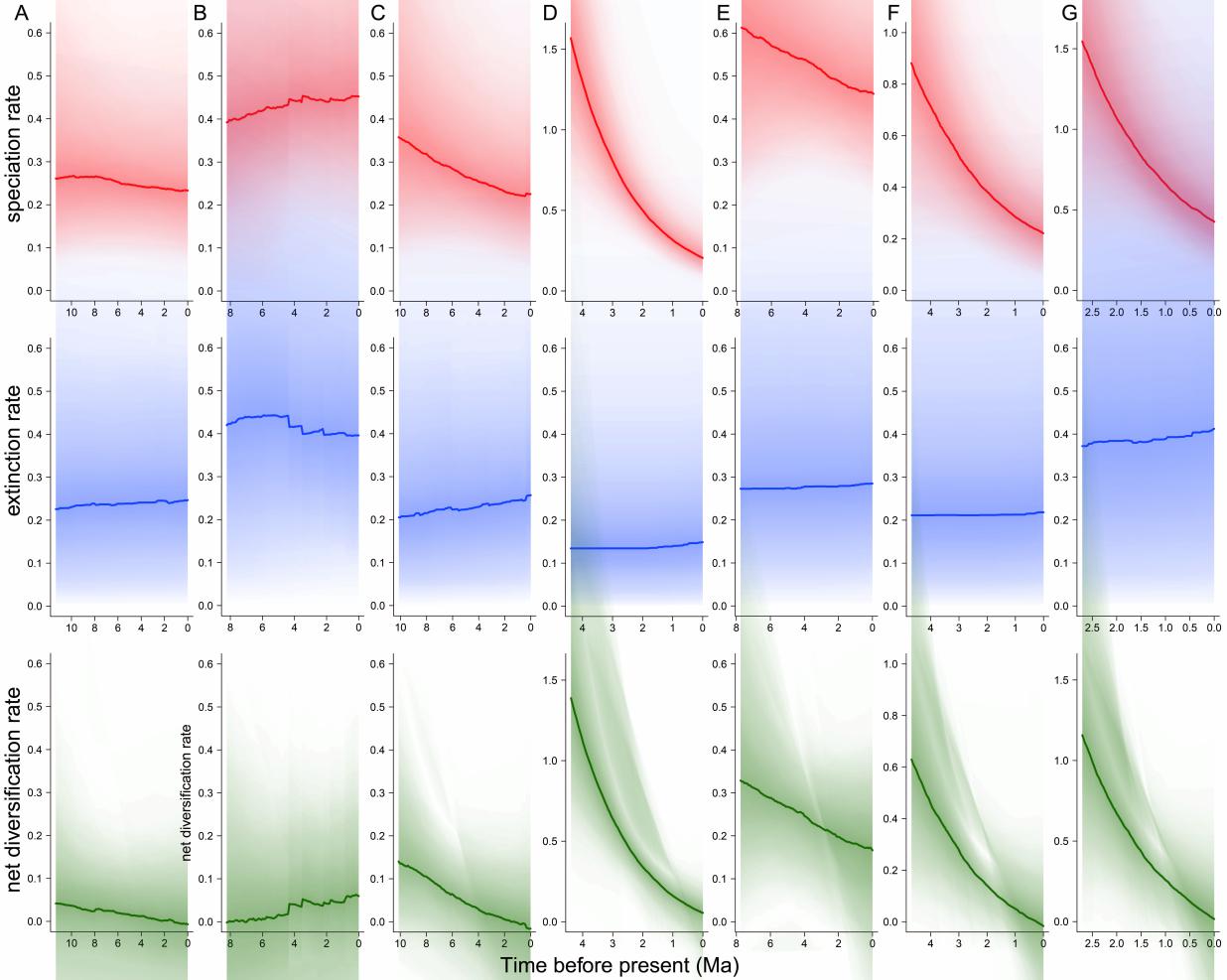
**

**Fig. S6.** Diversification rates of clades of *Impatiens* sect. *Impatiens*. (A) Clade I; (B) Clade II; (C) Clade III; (D) Clade IV; (E) Clade V; (F) Clade VI; (G) Clade VII.**
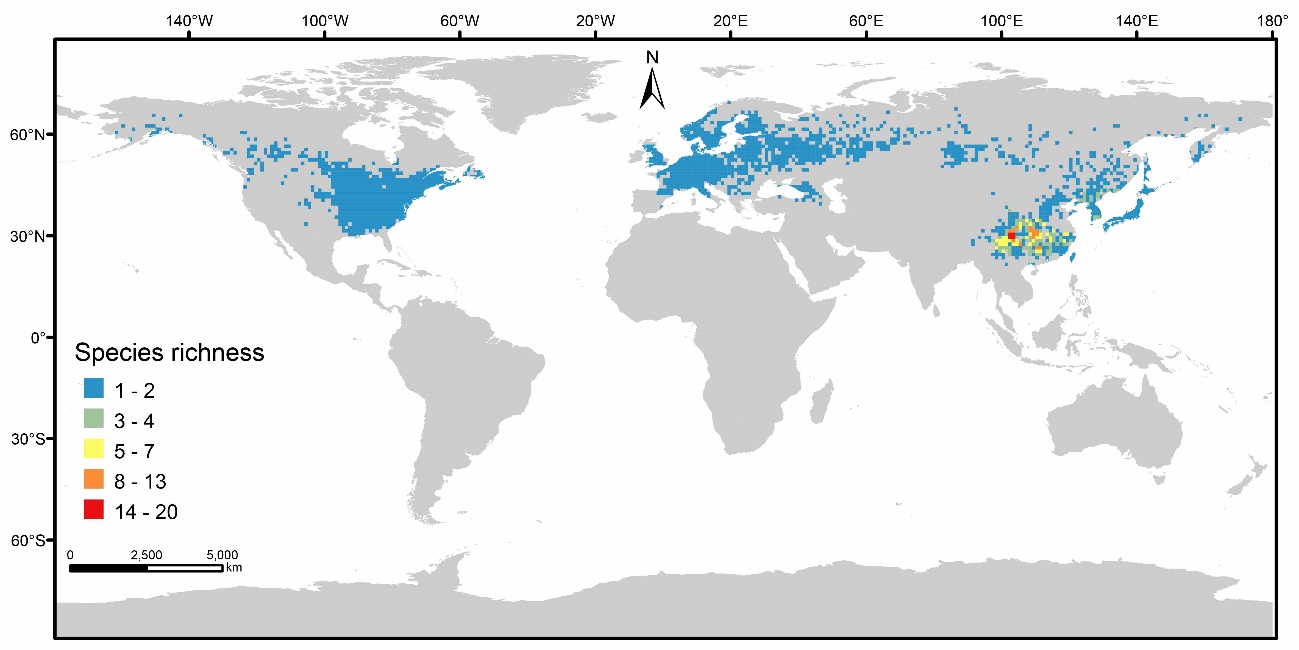
**

**Fig. S7.** Distribution pattern of the species richness of *Impatiens* sect. *Impatiens*. The map used in this study was downloaded from DIVA-GIS (http://www.diva-gis.org/Data).

**
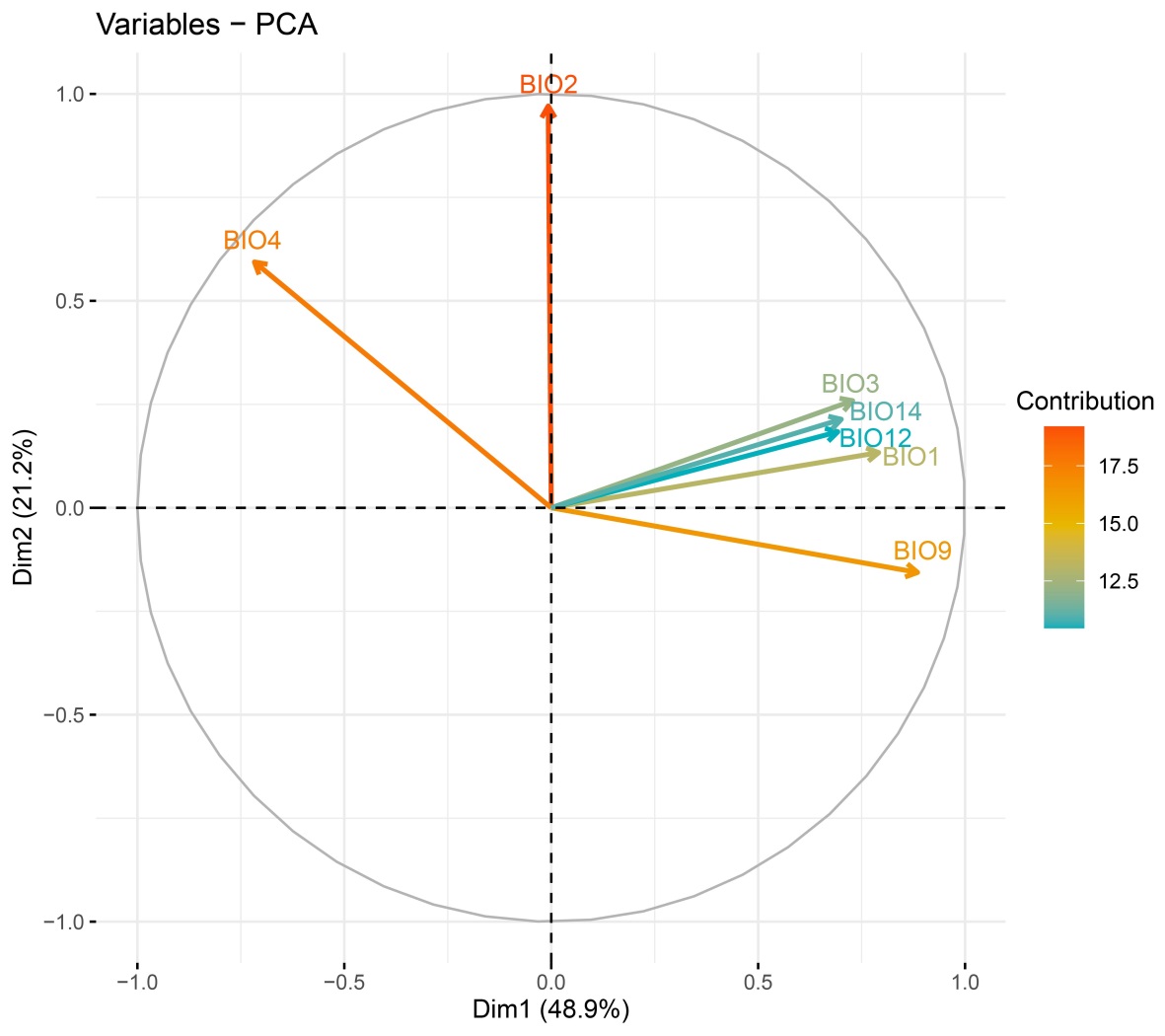
**

**Fig. S8.** Principal component analysis of the environmental factors relevant to *Impatiens* sect. *Impatiens*.

**
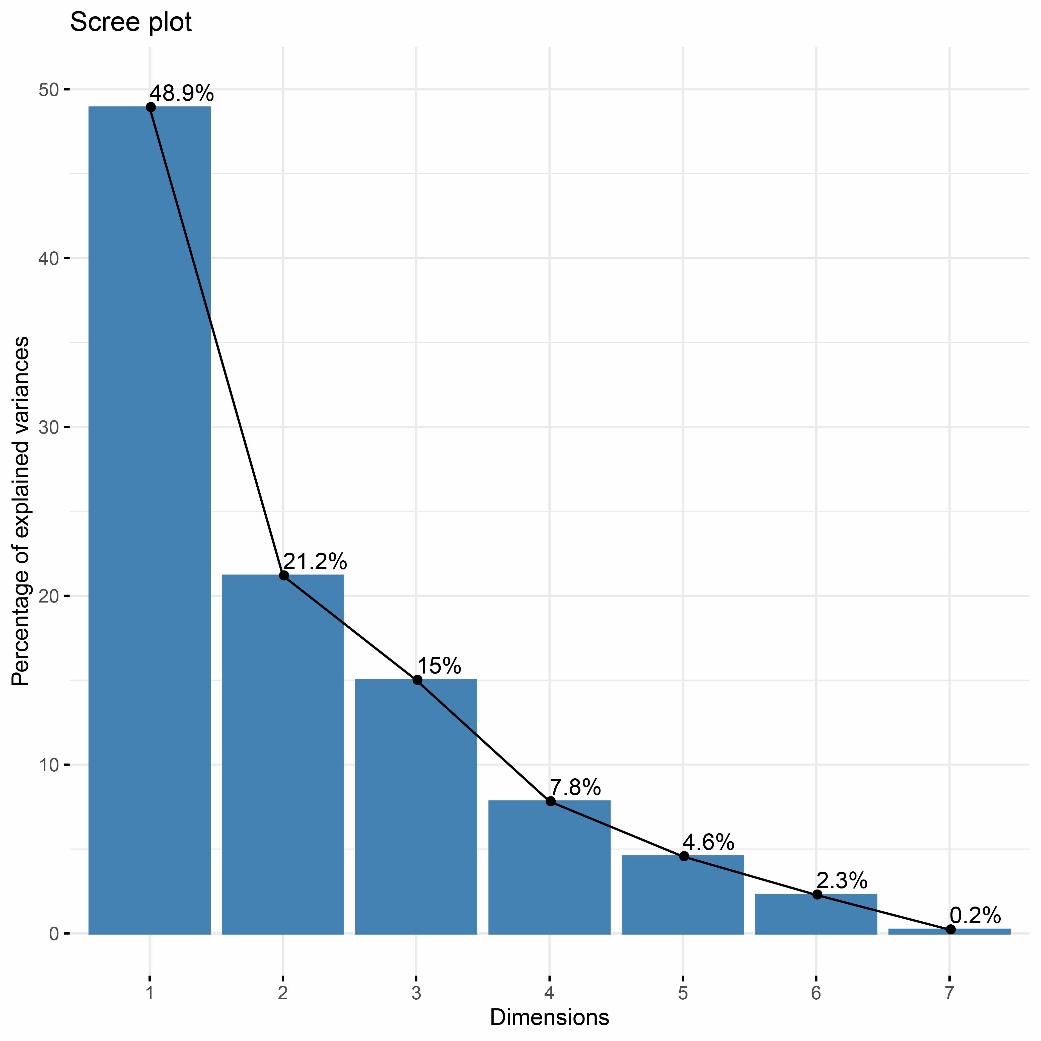
**

**Fig. S9.** Gravel diagram of principal component analysis.

**
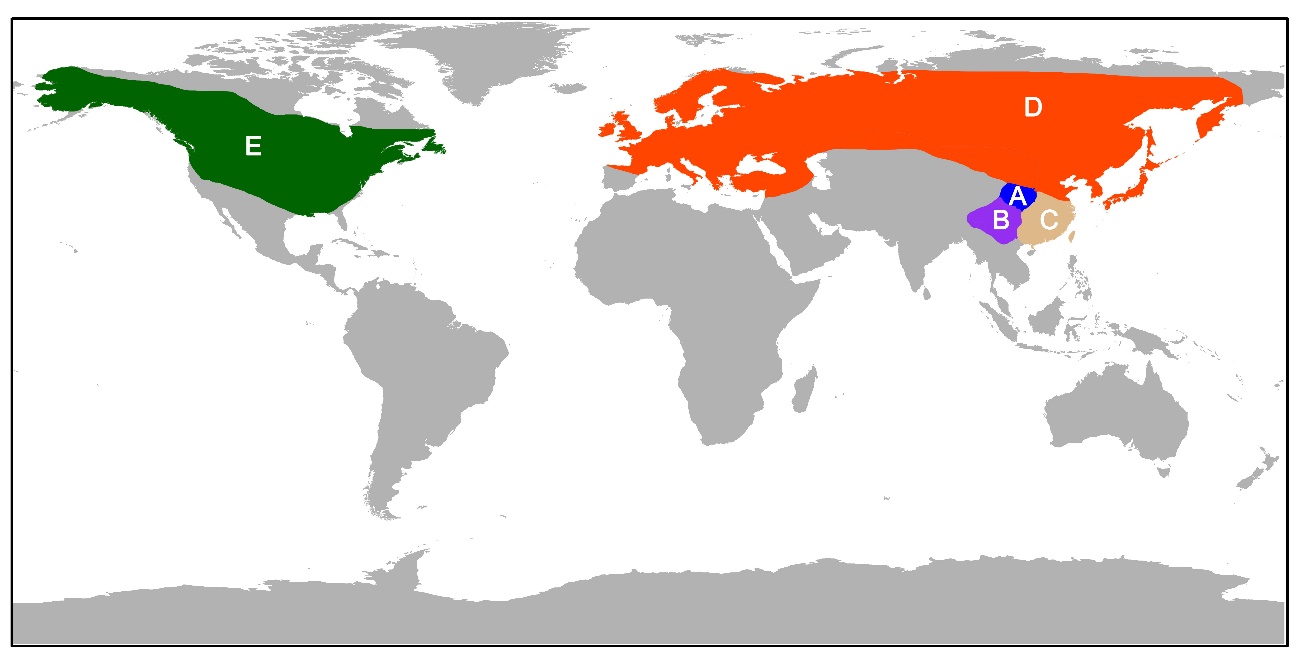
**

**Fig. S10.** Five geographical regions of *Impatiens* sect. *Impatiens* (A) Northwest China; (B) Hengduan Mountains; (C) Southeast China; (D) temperate Eurasia; (E) North America. The map used in this study was downloaded from DIVA-GIS (http://www.diva-gis.org/Data).
